# Tundra landform and vegetation productivity trend maps for the Arctic Coastal Plain of northern Alaska

**DOI:** 10.1038/sdata.2018.58

**Published:** 2018-04-10

**Authors:** Mark J. Lara, Ingmar Nitze, Guido Grosse, A. David McGuire

**Affiliations:** 1 Department of Plant Biology, University of Illinois Urbana-Champaign, Urbana, Illinois 61801, USA; 2 Institute of Arctic Biology, University of Alaska Fairbanks, Fairbanks, Alaska 99775, USA; 3 Alfred Wegener Institute Helmholtz Centre for Polar and Marine Research, Periglacial Research Unit, 14473 Potsdam, Germany; 4 Institute of Geography Science, University of Potsdam, 14476 Potsdam, Germany; 5 Institute of Earth and Environmental Science, University of Potsdam, 14476 Potsdam, Germany; 6 U.S. Geological Survey, Alaska Cooperative Fish and Wildlife Research Unit, University of Alaska Fairbanks, Fairbanks, Alaska 99775, USA

**Keywords:** Ecosystem ecology, Climate-change ecology

## Abstract

Arctic tundra landscapes are composed of a complex mosaic of patterned ground features, varying in soil moisture, vegetation composition, and surface hydrology over small spatial scales (10–100 m). The importance of microtopography and associated geomorphic landforms in influencing ecosystem structure and function is well founded, however, spatial data products describing local to regional scale distribution of patterned ground or polygonal tundra geomorphology are largely unavailable. Thus, our understanding of local impacts on regional scale processes (e.g., carbon dynamics) may be limited. We produced two key spatiotemporal datasets spanning the Arctic Coastal Plain of northern Alaska (~60,000 km^2^) to evaluate climate-geomorphological controls on arctic tundra productivity change, using (1) a novel 30 m classification of polygonal tundra geomorphology and (2) decadal-trends in surface greenness using the Landsat archive (1999–2014). These datasets can be easily integrated and adapted in an array of local to regional applications such as (1) upscaling plot-level measurements (e.g., carbon/energy fluxes), (2) mapping of soils, vegetation, or permafrost, and/or (3) initializing ecosystem biogeochemistry, hydrology, and/or habitat modeling.

## Background & Summary

Arctic polygonal tundra landscapes are highly heterogeneous, disproportionately distributed across mesotopographic gradients, varying in surficial geology, ground ice content, and soil thermal regimes^1,2^. The high density of ice wedges present in this low relief landscape facilitates subtle variations (~0.5 m) in surface microtopography, markedly influencing hydrology^3,4^, biogeochemistry^5–10^, and vegetation structure^11,12^. Fine-scale differences in microtopography have been shown to control a variety of key ecosystem attributes and processes that influence ecosystem function, such as snow distribution and depth^13^, surface and subsurface hydrology^13,14^, vegetation composition^2,11,12^, carbon dioxide and methane fluxes^5,6,15,16^, soil carbon and nitrogen content^17–19^, and an array of soil characteristics^18,20^. Despite the prominent control of microtopography and associated geomorphology on ecosystem function, land cover data products available to represent landforms across the Pan-Arctic are strikingly limited^21^. The relative absence of these key geospatial datasets characterizing permafrost lowlands, may severely limit our ability to understand local scale controls on regional to global scale patterns and processes^21^.

Datasets presented here were developed to investigate the potential local to regional controls on past and future trajectories of arctic tundra vegetation productivity^22^, inferred from spatiotemporal patterns of change in the Normalized Difference Vegetation Index (NDVI). We present two geospatial data products, (1) a 30 m resolution tundra geomorphology map, and (2) a decadal scale NDVI trend map (1999–2014), developed to represent the landform heterogeneity and associated productivity change across the Arctic Coastal Plain (ACP) of northern Alaska (~60,000 km^2^). We validated the tundra geomorphology map using 1000 reference sites, and evaluated the sensor bias used to develop the NDVI trend map. Produced geospatial datasets will be useful for an array of applications, some of which may include the (1) upscaling of plot-level measurements (e.g., carbon and energy fluxes), (2) mapping of soils, vegetation, or permafrost, and/or (3) initializing ecosystem biogeochemistry, hydrology, and/or habitat modeling.

## Methods

### Polygonal tundra geomorphology mapping

We focused this mapping initiative on the Arctic Coastal tundra region of northern Alaska, which stretches from the western coast along the Chukchi sea to the Beaufort coastal plains at the Alaskan-Canadian border (latitude: 68–71˚ N; longitude: 140–167˚ W). Two ecological landscape units (~60,000 km^2^), the Arctic peaty lowlands and the Arctic sandy lowlands were used to define the spatial extent of the ACP^23^. The region is dominated by continuous permafrost several hundred meters thick^24^. Permafrost ground ice content ranges from low in sandy lowlands to very high in peaty lowlands^23,25^, while the maximum active layer depth ranges from 20–120 cm^26^. These two arctic tundra regions (i.e., sandy and peaty lowlands) were specifically targeted in this analysis, due to their geomorphologic similarity to ~1.9 million km^2^ of tundra across the Pan-Arctic^27^. The tundra mapping approach described here will be useful for the development of comparable products across northern latitudes. Refer to the primary research article^22^, for detailed site descriptions.

### Image processing

Twelve cloud free Landsat 8 satellite images were acquired during the summers of 2013 and 2014, used in the tundra geomorphology classification (Table 1). All Landsat data products were downloaded from the United States Geological Survey (USGS) earth explorer web-based platform (https://earthexplorer.usgs.gov). We used only the 9 spectral bands provided by the Operational Land Imager (OLI) instrument for mapping, while ignoring the 2 additional Thermal Infrared Sensor (TIRS) bands due to defective optics in the infrared sensor^28^. Landsat 8 OLI spectral bands include (1) coastal/aerosol (Ultra blue), (2) blue, (3) green, (4) red, (5) near infrared (NIR), (6) shortwave infrared 1 (SWIR1), (7) shortwave infrared 2 (SWIR2), (8) panchromatic, and (9) cirrus. Prior to image mosaicking, reflectance values were normalized across satellite scenes, by calculating top-of-atmosphere reflectance^29^, which minimized the radiometric difference between images associated with varying atmospheric conditions, acquisition dates, and solar zenith angles^29^, while the Landsat Surface Reflectance Code (LaSRC) was used for atmospheric correction. Images were mosaicked within ArcGIS^TM^ 10.4 (ESRI).

### Image classification

**Figure 1:**
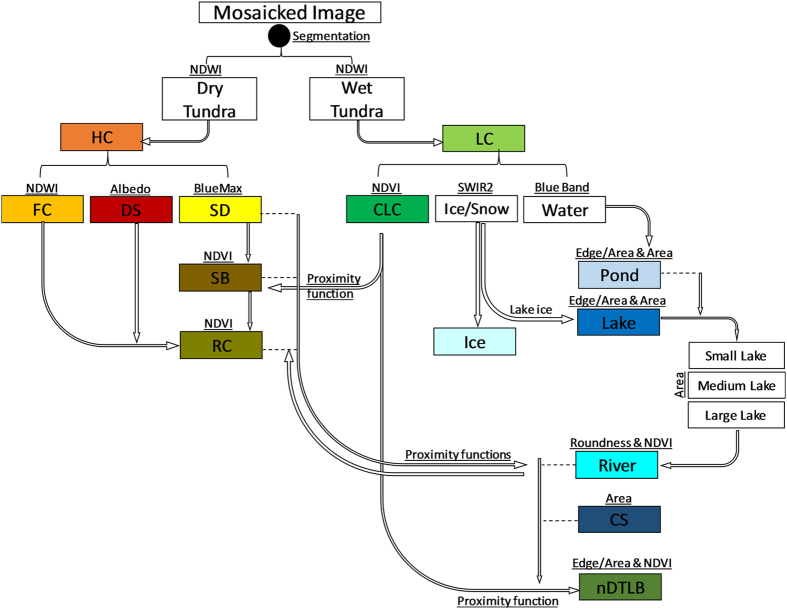
Simplistic schematic representation of the classification procedure used to map polygonal tundra geomorphology on the ACP. Underlined text represents Band, Area, Function, or Index thresholds used for assigning classes. Proximity functions are used to reclassify image objects based on distance from another geomorphic landform. See ‘Tundra Classification’ section for acronym definitions.

**Figure 2:**
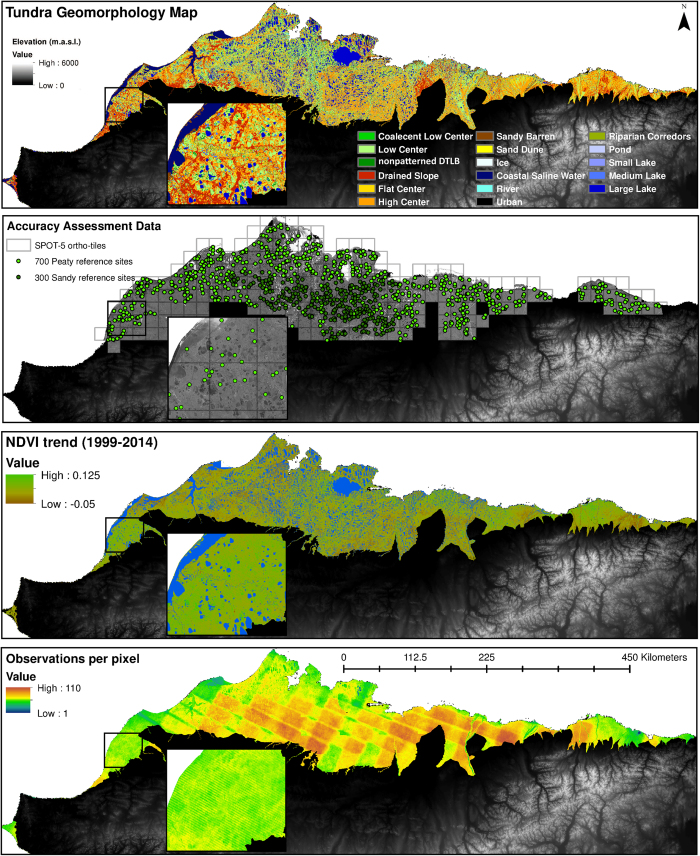
Geospatial datasets representing the heterogeneity in both landform and NDVI across the ACP of northern Alaska. The tundra geomorphology map (**a**) was validated with 1000 reference sites (700 and 300 in the Arctic Peaty Lowlands and Arctic Sandy Lowlands, respectively) using 249 SPOT-5 ortho-tiles (**b**), while the NDVI trend map (**c**) was developed using between 40 to 110 image observations per 30 m pixel (**d**).

We expand upon geomorphic mapping procedures developed for a subregion of the ACP of northern Alaska on the Barrow Peninsula (1800 km^2^)^5^, using a novel automated object based image analysis (OBIA) approach for tundra geomorphic mapping across the ACP (58,691 km^2^). The OBIA land cover classifier (eCognition™ version 9.1, Trimble) was parameterized using various rules, thresholds, spectral indices, and proximity functions using individual and combined spectral bands, spectral indices, and geometric object shapes/sizes (i.e., perimeter, area, roundness) and corresponding reference data (i.e., field/ground truth points and high resolution aerial/satellite imagery) to differentiate between geomorphic landforms (Fig. 1). Fifteen tundra geomorphic landforms were mapped at 30×30 m spatial resolution (Fig. 2a), including (qualitatively ranked from wet to dry), coastal saline water (CS), lakes (large:>90 ha, medium:≤90 and >20 ha, small:≤20 ha), rivers, ponds, coalescent low-center polygons (CLC), nonpatterned drained thaw lake basins (nDTLB), low-center polygons (LC), sandy barrens (SB), flat-center polygons (FC), riparian corridors (RC), high-center polygons (HC), drained slopes (DS), sand dunes (SD), ice/snow (Ice), and urban. Spectral indices used in image classification included Albedo^30^, Normalized Difference Vegetation Index (NDVI)^31^ ($\frac{\rho_{NIR-}\rho_{Red}}{\rho_{NIR}+\rho_{Red}}$), Normalized Difference Water Index (NDWI)^32^ ($\frac{\rho_{Green-}\rho_{NIR}}{\rho_{Green}+\rho_{NIR}}$), and BlueMax ($\frac{\rho_{Blue}}{\rho_{MaxDiff}}$), where MaxDiff refers to the maximum difference between all bands (1-9).

All pixels within the processed Landsat 8 image mosaic were aggregated into clusters or image ‘objects’ based on similar spectral properties of neighbouring pixels using multiresolution segmentation and spectral difference algorithms. These segmentation algorithms were parameterized to represent object characteristics such as shape, compactness, and spectral similarity. We split all image objects into two broad classes, wet tundra and dry tundra using NDWI thresholds, identified using landform specific field observations^5,15^. The following classification procedure (Fig. 1, Supplementary File 1), extracts all image objects from wet and dry tundra and reclassifies them into specific geomorphic landforms.

#### Wet Tundra Classification

We decomposed our classification of wet tundra into three steps, (1) extraction of CLC and nDTLB, (2) open water body differentiation, and (3) rectification of misclassifications. Initially, we differentiated CLC from all wet tundra objects using a low productivity (NDVI) threshold, which was associated with sparse vegetation cover and the presence of open water. Although, both CLC and nDTLB are found in aquatic to wet environments, we differentiated CLC from nDTLB landforms using the characteristically high NDVI values of nDTLB^5,15^ and morphological features. Due to the rapid formation of nDTLB following lake drainage^33^, this young geomorphic landform often contains a relatively large non-polygonal surface area^34^ (i.e., limited effects of ice aggregation and heaving processes associated with microtopographic variability), thus we use a moderate edge to area ratio and high NDVI threshold for nDTLB feature extraction.

All unvegetated open water pixels were extracted using a low-moderate blue band threshold (Fig. 1). A spectral difference segmentation algorithm, was looped 5x to iteratively combine all neighbouring open water objects with similar spectral properties. This object merging process enabled the identification of each spatially isolated water body (i.e., lake, pond, or river), where structural properties such as area, perimeter, or edge (i.e., perimeter) to area ratio can be used to differentiate waterbodies. Therefore, we defined CS, lakes, and ponds using structural properties, area and edge to area ratio. Water bodies were decomposed into CS (>100,000 ha) large lakes (≤ 100,000 > 90 ha), medium lakes (≤ 90 > 20 ha), small lakes (≤ 20 >1 ha), and ponds (≤ 1 ha). The 100,000 ha area threshold was used to define CS to avoid large lake misclassification errors, as Teshekpuk Lake (70.61˚ N, −153.56˚ W), has an area of ~83,000 ha. Due to misclassifications of ponds as lakes, associated with the high interconnectivity between irregularly structured open water objects, we used a low edge to area ratio on lakes, to ensure accurate classification of ponds. Rivers were differentiated from all open water objects using a NDVI threshold and a ‘roundness’ function. Integrating both approaches successfully extracted rivers, as high NDVI thresholds were used to differentiate open water from vegetated aquatic standing water objects, and low roundness values identified the characteristic elongated and meandering structure of rivers. Despite the late summer image acquisition dates used in this classification (Table 1), ice/snow image objects identified using high SWIR2 thresholds, were found in large lakes or adjacent to steep topographic gradients such as river valleys or near a snow fence. All ice/snow objects that occurred on lakes were reclassified as lake area, while the remaining ice/snow was reclassified as Ice.

Although, classification functions developed for wet tundra performed well, the majority of misclassifications were associated with the relatively course spatial resolution object patch size (30 m). To rectify these misclassifications, we used neighborhood or proximity functions to develop relationships between nearby geomorphic landforms using spectral and structural parameters for nDTLB, CLC, pond, and lakes. For example, nDTLB was often misclassified as CLC or pond, occurring near lake perimeters. Because aquatic-wet landforms occurring near lake perimeters are typically represented by nDTLB, having recently formed after partial or complete lake drainage, we reclassified older landforms such as CLC and ponds adjacent to lakes as nDTLBs. All remaining unclassified wet tundra objects that did not meet the criteria for nDTLB, CLC, pond, river, CS, or lakes in wet tundra were classified as LC (i.e., dominant wet geomorphic landform).

#### Dry Tundra Classification

We differentiated landforms in dry tundra following two steps, (1) threshold identification and extraction of FC and RC, and (2) rectification of misclassifications. A series of reference sites identified from ground based observations and/or oblique aerial photography were used to define NDWI and NDVI thresholds needed to extract FC and RC, respectively. These two geomorphic landforms were difficult to classify due to the similarity in vegetation composition and surface hydrology. However, we were able to differentiate between these two landforms, as FC was slightly higher in surface wetness, associated with the 2 fold difference in trough area relative to HC^5^. The high variability in NDVI of shrub canopies in RC relative to other landforms, made RC difficult to extract. Nevertheless, because RC typically occurred near riverine environments, we used both a low-moderate NDVI threshold and a proximity function adjacent to rivers to extract RC. Sand and gravel objects were easily extracted using a high BlueMax threshold. All lightly vegetated wet-moist sand and gravel objects were classified as SB using a moderate-high NDVI threshold, whereas drier sand and gravel objects were classified as SD. Due to the use of sand and gravel in the development of urban infrastructure such as roads and buildings, automated procedures initially classified these feature as SD, as they had a similar spectral signature. However, we manually reclassified SD as Urban near native Alaskan villages and oil drilling platforms (i.e., near Prudhoe Bay). Although, we made significant progress with the development of classification procedures for Urban landforms using spectral patterns and geometric structures, we abandoned this development due to the relatively limited area impacted by urban infrastructure across the ACP. Additionally, DS was extracted using a high albedo threshold, as this landform was very dry and often dominated by lichen plant communities, which are highly reflective^15^. Similar to misclassifications associated with object patch size identified in wet tundra, we found analogous misclassifications of SB near rivers as CLC and ponds. Therefore, we reclassified CLC and pond classes that were adjacent to rivers as SB. All remaining unclassified dry tundra objects not classified as DS, FC, RC, SB, SD, or Urban were classified as HC (i.e., dominant dry geomorphic landform).

### Decadal scale NDVI trend analysis

Following the approach of Nitze & Grosse^35^, the NDVI trend map (Fig. 2d) was computed using all available imagery collected from the Landsat sensors Thematic Mapper (TM), Enhanced Thematic Mapper+ (ETM+), and Observing Land Imager (OLI), acquired between July 1st and August 30th (i.e., peak growing season) of 1999-2014, across the ACP. We excluded imagery preceding 1999 due to the paucity of image acquisition and limited coverage across the ACP. All surface reflectance data used to derive this product were downloaded as radiometrically and geometrically terrain-corrected product from the USGS EROS Science Processing Architecture interface (https://espa.cr.usgs.gov). The ‘FMask’ algorithm^36^ was used to detect and mask out all non-valid data, such as clouds, shadows, snow/ice, and nodata pixels. For each pixel, linear trends of NDVI were calculated using the non-parametric Theil-Sen linear regression method, which calculates the median of all possible slopes across every point in time^37,38^. The Theil-Sen regression is robust against outliers and outperforms least-squares regression in remote sensing data^39^. Each pixel within the NDVI trend map was based on a total of 40-110 Landsat images (Fig. 2c) for the Theil-Sen slope calculation. The final NDVI trend product was spatiotemporally similar to coarser resolution products^40,41^ identifying heterogeneous patterns of greening and browning across the ACP of northern Alaska.

## Data Records

The presented ACP tundra geomorphology map (Data Citation 1), NDVI trend map (Data Citation 2) and all spatial and climate data used in Lara *et al*.^22^ are archived at the Scenarios Network of Alaska and Arctic Planning (SNAP) data portal. These spatial data products were clipped to the ACP domain and formatted as geotiff rasters.

Although the tundra geomorphic map was developed using OBIA which clusters spectrally similar nearby pixels into objects, the final map was resampled at the original 30×30 m pixel resolution and presented as a single-band raster (Fig. 2a). The map attribute table includes the following data columns: geomorphic landform (i.e., sand dune, low-center polygon), area (km^2^), and soil moisture regime (SMR). In addition, a color palette file (.clc) is provided to reproduce map (Fig. 2a). The annotated functions and code used for the classification of tundra landforms within eCognition™ v. 9.1, are made available in the supplementary information. All threshold values were replaced with qualitative ranges (i.e., low, low-moderate, moderate, moderate-high, or high) as reflectance values and image statistics will vary between scenes, thus user specific refinement will be required. Further, it is important to note that the classification procedure developed here has only been evaluated in lowland arctic tundra ecosystems and misclassifications may arise if applied in dissimilar tundra environments. For example, we applied the developed classification procedure to higher elevation drier hillslope tundra, south of the ACP, finding the rate of misclassification to increase, as algorithms/functions were initially developed explicitly for polygonal tundra similar to the ACP of northern Alaska. To include different tundra landforms with different vegetation, hydrology, and soil characteristics, further development will be required.

The NDVI trend map is presented as a four-band raster (Fig. 2d). Band 1 represents the decadal scale rate of change or slope calculated by the Theil-Sen regression. Band 2 represents the intercept or the NDVI data scaled to the year 2014. While, Band 3 and 4 are the upper and lower 95% confidence intervals of the slope of each individual pixel.

## Technical Validation

### Tundra Geomorphology Map

To validate the tundra geomorphology map, we used an array of oblique aerial/ground based photography and 249 high resolution Satellite Pour l’Observation de la Terre 5 (SPOT-5) orthorectified image tiles covering >80% of the ACP, provided by the Geographic Information Network of Alaska (GINA, gina.alaska.edu). A stratified random sampling of 700 and 300 reference sites in the Arctic peaty lowlands and Arctic sandy lowlands^23^, respectively (Fig. 2b), were used for the accuracy assessment. At each of the 1000 sites, we manually generated a reference dataset for geomorphic landforms using high resolution products (Table 2, Supplementary Table 2). This process has been used previously^5^, identifying 95.5% agreement between reference sites (e.g., geomorphology) generated from satellite platforms relative to that observed on the ground.

Overall map accuracy was 75.7% and Cohens Kappa was 0.725 (Table 3), suggesting the strength of agreement between the independent validation (i.e., reference) dataset and classification to be good to very good^42,43^. Our map had relatively high user and producer accuracies (Table 3), with the exception of FC, which had a producer accuracy of 40.5%. This relatively low producer accuracy was expected as we had difficulties identifying unique spectral and structural characteristics of FC that that differed from HC. This identification challenge was highlighted in the accuracy assessment, as 64% of misclassified FC were classified as HC, similar to other tundra geomorphic classifications^5^. The relatively low producer accuracies for FC, CLC, and DS are likely associated with the challenge of decomposing a complex continuously evolving geomorphic landscape^13,33,44,45^ such as the Arctic tundra into discrete landform units. Despite these difficulties, our accuracy assessment suggests the tundra geomorphology map well represented the spatial distribution and heterogeneity of tundra landforms. We present for the first time, a detailed framework for characterizing arctic tundra landforms across the Pan-Arctic.

### NDVI Trend Map

**Figure 3:**
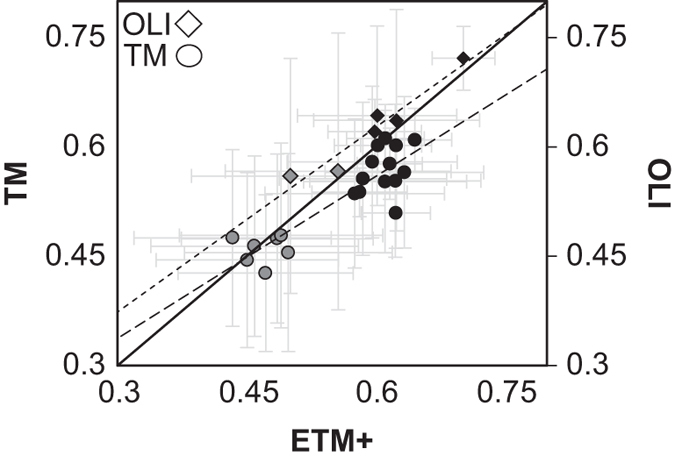
Estimate of NDVI bias between Landsat sensors, represented at three subregions of northern Alaska. Each point represents the mean (±standard deviation) of NDVI for a single year and subregion. Circles and diamonds represent TM and OLI plotted against ETM+. Grey points represent means from polygonal tundra within the ACP, while black points represent more southerly sites (i.e., foothills of the Brooks Range and Selawik lowlands). Dashed and dotted lines represent trend lines for TM and ETM+ and OLI and ETM+, respectively. The solid black line indicates a 1:1 line.

We evaluated the potential sensor bias between TM, ETM+, and OLI, used to derive the NDVI Trend Map by comparing the mean value for each pixel, year, and sensor computed from three different locations in northern Alaska (Fig. 3). Each location was composed of 40,000 pixels (~36 km^2^). The three centroids of each location are found in the (1) Arctic sandy lowlands of the ACP (longitude: −154.50, latitude: 70.09), (2) foothills of the Brooks Range on the North Slope (longitude: -159.61, latitude: 66.60), and (3) Selawik lowlands in northwestern Alaska (longitude: −152.92, latitude: 69.29). Minor discrepancies were to be expected between sensor platforms as the images were not acquired at the same time or day.

We identified minor NDVI sensor biases between sensors (Fig. 3), while sensor specific NDVI distributions were consistent. Most of the data used to generate the NDVI trend map was acquired from the ETM+ sensor, as it was available throughout our data acquisition window (i.e., 1999–2014), whereas data from TM and OLI were only available between 2005-2011 and 2013-2014, respectively. Mean sensor bias estimates for TM and OLI across all subregions of Alaska, indicate NDVI to be slightly under- and overestimated relative to ETM+, though the variability was high within each year and subregion (Fig. 3). The minor sensor bias identified here, was similar to that identified across North American high latitude terrestrial ecosystems^41^. Although, it is likely that sensors are slightly positively (OLI) and negatively (TM) biased with respect to ETM+ across northern Alaska, sensor calibrations appeared to well represent the tundra subregion on the ACP (Fig. 3). NDVI values from both TM and OLI sensors clustered above and below the 1 to 1 line for the subregion on the ACP (Fig. 3), suggesting NDVI data was not positively or negatively skewed between sensors. A slight positive linear NDVI bias (+0.00063) was detected across all sensor data, suggesting a satisfactory agreement between sensors used to compute NDVI on the ACP.

## Additional information

**How to cite this article**: Lara, M. J. *et al.* Tundra landform and vegetation productivity trend maps for the Arctic Coastal Plain of northern Alaska. *Sci. Data* 5:180058 doi: 10.1038/sdata.2018.58 (2018).

**Publisher’s note**: Springer Nature remains neutral with regard to jurisdictional claims in published maps and institutional affiliations.

## Supplementary Material



Supplementary Information

## Figures and Tables

**Table 1:** Mosaicked Landsat scenes used to create the tundra geomorphology map.

**Product ID**	**Sensor**	**Satellite**	Year*	Month*	Day*
LC80690112013249LGN00	OLI/TIRS	Landsat 8	2013	Sept.	5
LC80720112013254LGN00	OLI/TIRS	Landsat 8	2013	Sept.	10
LC80740112014191LGN00	OLI/TIRS	Landsat 8	2014	July	9
LC80770102013193LGN00	OLI/TIRS	Landsat 8	2013	July	11
LC80770112013193LGN00	OLI/TIRS	Landsat 8	2013	July	11
LC80790102013191LGN00	OLI/TIRS	Landsat 8	2013	July	9
LC80800102014217LGN00	OLI/TIRS	Landsat 8	2014	Aug.	4
LC80800112014249LGN00	OLI/TIRS	Landsat 8	2014	Sept.	5
LC80820122013244LGN00	OLI/TIRS	Landsat 8	2013	Aug.	31
LC80830102014222LGN00	OLI/TIRS	Landsat 8	2014	Aug.	9
LC80830112014190LGN00	OLI/TIRS	Landsat 8	2014	July	8
LC80840122013194LGN00	OLI/TIRS	Landsat 8	2013	July	12

*Acquisition date.

**Table 2:** Example of the reference dataset generated to validate the tundra geomorphology map.

Ecological Landscape	Landform	Latitude	Longitude
Arctic Peaty Lowland	non-patterned Drained Thaw Lake Basin	71.236855	−156.3785131
Arctic Peaty Lowland	High-center polygon	71.210642	−156.4676783
Arctic Peaty Lowland	Pond	71.191358	−156.3469935
Arctic Peaty Lowland	River	70.181085	−147.2617363
Arctic Peaty Lowland	Riparian corridor	70.165456	−148.4265964
Arctic Sandy Lowland	Lake	70.166375	−154.2431094
Arctic Sandy Lowland	Drained slope	70.160143	−153.6316156
Arctic Sandy Lowland	Sand dune	70.3493	−152.7590333
The complete (1000 point) reference dataset can be found in the Supplementary Table 2.			

**Table 3:** Accuracy assessment represented as a confusion matrix.

	**Reference Sites**													
**Geomorphic type**	**SB**	**SD**	**RC**	**DS**	**HC**	**FC**	**LC**	**nDTLB**	**CLC**	**Pond**	**River**	**Lake**	**CS**	**User accuracy**
*Classification*														
SB	**12**				2	2					1			63%
SD	3	**12**			2									71%
RC			**4**				1							80%
DS				**50**	19	4								69%
HC				35	**215**	30	22	2						70%
FC					11	**32**	3							71%
LC			1	6	34	11	**152**	5	7			2		70%
nDTLB					3		16	**53**	1		1	2		70%
CLC							2	2	**15**			2		71%
Pond										**18**				100%
River	2							2	2		**10**			63%
Lake							1	1				**156**		99%
CS												1	**28**	97%
**Producer accuracy**	71%	100%	80%	55%	75%	41%	77%	82%	56%	100%	83%	96%	100%	1000
**Overall accuracy**	**76%**													
**Cohens Kappa**	**0.73**													
Bolded diagonal values within the matrix represent correctly identified pixels, where User and Producer accuracies are presented on the right vertical axis and bottom horizontal axis.														
