# Supplementary material for: Tundra landform and vegetation productivity trend maps for the Arctic Coastal Plain of northern Alaska
Source: Sci Data. 2018 Apr 10;5:180058. doi: 10.1038/sdata.2018.58 (PMC5892374; doi:10.1038/sdata.2018.58)
Supplement: Supplementary Information [file sdata201858-s2.docx]

# **Tundra landform and vegetation productivity trend maps for the Arctic Coastal Plain of northern Alaska**

Mark J. Lara^*^, Ingmar Nitze, Guido Grosse, A. David McGuire

**Supplementary File 1: Rules, thresholds, functions developed in eCognition 9.1 for the classification of tundra landforms on the ACP of northern Alaska.**

Trimble eCognition polygonal tundra mapping code

Mapped Classes (**bolded** are final classes, unbolded are used to derive final classes, referred to in rules below):

1. Wet tundra
   1. **Coalescent Low Center (CLC)**
   2. **Low Center (LC)**
   3. **Nonpatterned Drained Thaw Lake Basins (nDTLB)**
2. Open Water
   1. **Coastal Saline Water (CS)**
   2. Lake
      1. **Small Lake (sLake)**
      2. **Medium Lake (mLake)**
      3. **Large Lake (lLake)**
   3. **Pond**
   4. **River**
3. Dry tundra
   1. **Drained Slope (DS)**
   2. **High Center (HC)**
   3. **Flat Center (FC)**
   4. **Sandy Barren (SB)**
   5. **Sand Dune (SD)**
   6. **Riparian Corridor (RC)**
4. Other
   1. **Ice**
   2. **Urban**
5. NoData

----------------------------------------------------------------------------------------------------------------------------

Process: Main: (*italic text comments on the use and purpose of the threshold/rule/function*)

- 1. Geomorphic Characterization *(The following functions did a good job segmenting polygonal tundra at 30 m resolution using Landsat 8 imagery)*

1. multiresolution segmentation: 25 [shape:0.5 compct.:0.9] creating 'New Level'
2. spectral difference segmentation: at New Level: spectral difference 35
   1. Wet Tundra *(contains all wet, aquatic, and open water image objects)*
3. assign class: unclassified with NDWI <= [*moderate*] at New Level: Wet Tundra (*threshold that assumes all wet to open water objects. The initial identification of wet vs dry tundra is important to correctly identify. Suggest using ground truth and/or reference datasets)*
4. assign class: Wet Tundra at New Level: LC *(convert wet objects to Low center as default, as this is the dominant landform)*
5. assign class: LC with NDVI >= [*moderate*] at New Level: CLC *(derives CLC from LC)*
6. assign class: CLC, LC with Blue Band <= [low-*moderate*] at New Level: Open Water *(threshold to extract all aquatic non vegetated objects from Wet Tundra)*
7. spectral difference segmentation: 5x: Open Water at New Level: spectral difference 250 *(combines all nearby open water objects based on similar spectral properties to reduce number of objects within isolated water bodes such as ponds and lakes)*
8. assign class: Open Water at New Level: Pond *(convert all open water objects to pond by default. This approach is used to ensure there are no left over open water objects that do not meet the criteria defined below)*
9. assign class: Pond with Area >= 1 ha and Edge/Area <= [*low*] at New Level: Lake *(defines and extracts lakes by “area” and “edge to area ratio” to avoid misclassification of ponds as lakes that contain a sequence of interconnected image objects with irregular shapes yielding to a high area)*
10. assign class: Lake with Area >= 100000 ha at New Level: CS *(defines and extracts CS by “area”. This function finds large water bodies indicative of marine waters, but may need to be adjusted if very large lakes are within your scene or the image extent. See Teshekpuk lake example in the manuscript main text)*
11. assign class: CS, Lake with Area >= 90 ha at New Level: lLake *(defines and extracts lLakes by “area”)*
12. assign class: CS,Lake with Area >= 20 ha at New Level: mLake *(defines and extracts mLakes by “area”)*
13. assign class: CS, Lake with Area >= 1 ha at New Level: sLake *(defines and extracts sLakes by “area”)*
14. assign class: lLake, mLake, sLake, Lake, Pond with Roundness >= [*high*] and NDVI >= [*moderate*] at New Level: River *(extracts rivers as a function of roundness and greenness. NDVI is added here to differentiate rivers from vegetated standing water that take the shape of a river or stream)*
15. assign class: CS, lLake, mLake, sLake, Pond with NDVI <= [*high*] and Edge/Area<=[*moderate]* at New Level: nDTLB *(nDTLBs often occupy large areas not small isolated landscape features so a cutoff using edge to area is used with a vegetation threshold to differentiate from water bodies. Despite the high productivity and associated high NDVI characteristic of this landform, we use a low NDVI threshold as nDTLB may be seasonally flooded and may appear spectrally similar to water bodies, but the NDVI signal differentiates waterbodies from nDTLB.)*
16. grow region: CS at New Level: <- River <not found> = 0 *(occasionally the spectral reflectance patterns, near the shoreline causes misclassifications of CS as river. Thus, we “grow” or reclassify nearby CS objects into river objects)*
    1. Lake Boarder
17. grow region: nDTLB at New Level: <- CLC <not found> = 0 *(nDTLB are young landforms and CLCs are old, therefore they are rarely found adjacent to one another. We use this rule to reclassify nDTLBs near CLC)*
18. assign class: CLC, LC with Rel. border to Lake <= 0.95 and Rel. border to Lake >= 0.1 at New Level: nDTLB *(reclassified CLC and LC near lake margins as nDTLB, as nDTLB were occasionally misclassified due to image object patch size error.)*
    - assign class: Pond with Rel. border to Pond <= 0.35 and Rel. border to Lake >= 0.1 at New Level: nDTLB *(similar logic as above point, but for ponds)*
    1. Lake Ice
19. assign class: Wet Tundra, unclassified, Open Water with SWIR2 >= [*high*] at New Level: Ice *(this function is rarely implemented as all imagery used was acquired between July and September when most of the terrestrial surface ice/snow had already melted.)*
    - [assign class: Ice at New Level: OpenWater] *(after ice was confirmed that it was over open water we looped these objects back through the “Wet Tundra” rules for classification of specific water bodies [2e-2n])*
20. Dry Tundra *(contains all dry and moist image objects)*
21. assign class: unclassified with NDWI > [*moderate*] at New Level: Dry Tundra (T*hreshold that assumes all dry objects. The initial identification of wet vs dry tundra is important to correctly identify. The hierarchical tundra classification that follows will be significantly impacted. Suggest using ground truth and/or reference datasets)*
22. assign class: Dry Tundra at New Level: HC *(convert all Dry Tundra objects to HC as the default)*
23. assign class: Dry Tundra, HC with NDWI >= [moderate-high] and NDWI <= [moderate-high] at New Level: FC *(need to use ground control sites to fine-tune the extraction of FC)*
24. assign class: Dry Tundra, HC, FC with Albedo >= [moderate-high] at New Level: DS *(This landform was very reflective as it was dry and dominated by lichen communities, which were found to correspond well with DS)*
25. assign class: Wet Tundra, Dry Tundra, CLC, DS, HC, FC, LC, nDTLB, Open Water with BlueMax >= [high] at New Level: SD *(nicely extracts regions that are sandy and/or gravely. All landforms are included in this function to catch any potential misclassifications)*
26. assign class: SD with NDVI >= [*moderate-high*] at New Level: SB *(NDVI is able to differentiate SB from SDs)*
27. grow region: SB at New Level: <- CLC *(reclassifies CLC adjacent to SB as SB. This function cleans up errors associated with patch misclassifications near SB)*
28. assign class: CLC, LC with Rel. border to River >= 0.05 at New Level: SB *(reclassifies CLC and LC adjacent to rivers as SB. This function cleans up errors associated with patch misclassifications near SB)*
    - grow region: loop: SB at New Level: <- CLC *(loops the reclassification of CLC adjacent to SB as SB. Cleans up errors associated with misclassification near SB)*
29. grow region: loop: River at New Level: <- Pond *(reclassifies ponds adjacent to river as river. Rivers channels begin to narrow moving inland and patch misclassifications increase in the form of river misclassified as ponds.)*
30. assign class: Pond with Rel. border to SB >= 0.01 at New Level: River *(reclassifies ponds adjacent to SB as river. Similar to the above function, as rivers channels narrow beyond ~30 m width misclassifications increase. This function cleans up errors associated with patch misclassification of SB as ponds)*
31. assign class: CLC with Rel. border to River >= 0.2 at New Level: SB *(reclassifies CLC adjacent to river as SB. Rivers channels narrow moving inland, thus misclassifications increase associated with image patch size.)*
32. assign class: SB, SD with NDVI <= [*moderate*] at New Level: RC *(reclassifies SB and SD with prescribed NDVI as RC. Identifies sparse shrubs indicative of RC growing on SB/dunes)*
33. assign class: DS, FC, HC, SB, SD with NDVI >= [*low-moderate*] and NDVI <=[moderate] at New Level: RC *(reclassifies DS, FC, HC, SB, SD with prescribed NDVI as RC. Identifies dense shrub cover that make it difficult to identify polygonal tundra geomorphology, typically near riparian habitats)*
34. NoData Background *(many methods can be used, any function to find values that are null will work)*
35. assign class: Wet Tundra, Dry Tundra, NoData, Open Water, unclassified with Albedo <= 0 at New Level: NoData
36. Merge *(combines all neighboring objects into individual classes to reduce file size)*
37. merge region: CLC at New Level: merge region
38. merge region: LC at New Level: merge region
39. merge region: nDTLB at New Level: merge region
40. merge region: DS at New Level: merge region
41. merge region: FC at New Level: merge region
42. merge region: HC at New Level: merge region
43. merge region: SB at New Level: merge region
44. merge region: SD at New Level: merge region
45. merge region: CS at New Level: merge region
46. merge region: Lake at New Level: merge region
47. merge region: Pond at New Level: merge region
48. merge region: River at New Level: merge region
49. merge region: RC at New Level: merge region
50. merge region: Urban at New Level: merge region

Supplementary Table 2: One-thousand point reference dataset generated using high resolution aerial and satellite imagery to validate the tundra geomorphology map.

| **Ecological Landscape** | **Landform** | **Latitude** | **Longitude** |
| --- | --- | --- | --- |
| Arctic Peaty Lowland | non-patterned Drained Thaw Lake Basin | 71.23685526 | -156.3785131 |
| Arctic Peaty Lowland | High-center polygon | 71.21064193 | -156.4676783 |
| Arctic Peaty Lowland | Pond | 71.19135817 | -156.3469935 |
| Arctic Peaty Lowland | High-center polygon | 71.181325 | -156.666146 |
| Arctic Peaty Lowland | High-center polygon | 71.18051471 | -156.0699113 |
| Arctic Peaty Lowland | Low-center polygon | 71.17085284 | -156.8198123 |
| Arctic Peaty Lowland | Low-center polygon | 71.15948007 | -156.6037783 |
| Arctic Peaty Lowland | non-patterned Drained Thaw Lake Basin | 71.13740862 | -156.8034174 |
| Arctic Peaty Lowland | Drained slope | 71.13705996 | -156.9119976 |
| Arctic Peaty Lowland | Drained slope | 71.13670228 | -156.7770417 |
| Arctic Peaty Lowland | Low-center polygon | 71.08713235 | -156.2764537 |
| Arctic Peaty Lowland | Lake | 71.08463425 | -155.6195661 |
| Arctic Peaty Lowland | Low-center polygon | 71.07225494 | -157.1680188 |
| Arctic Peaty Lowland | Low-center polygon | 71.07059335 | -156.2491311 |
| Arctic Peaty Lowland | High-center polygon | 71.07022569 | -157.093039 |
| Arctic Peaty Lowland | High-center polygon | 71.06501637 | -155.1197408 |
| Arctic Peaty Lowland | Low-center polygon | 71.06218897 | -156.961219 |
| Arctic Peaty Lowland | Coalecent low-center polygon | 71.05431794 | -155.1588828 |
| Arctic Peaty Lowland | Low-center polygon | 71.02896586 | -155.8633919 |
| Arctic Peaty Lowland | Coalecent low-center polygon | 71.01400735 | -156.792821 |
| Arctic Peaty Lowland | Flat-center polygon | 71.00290572 | -156.8585227 |
| Arctic Peaty Lowland | non-patterned Drained Thaw Lake Basin | 70.99696697 | -156.4121189 |
| Arctic Peaty Lowland | Low-center polygon | 70.98162601 | -156.0403792 |
| Arctic Peaty Lowland | Drained slope | 70.97987194 | -156.4706415 |
| Arctic Peaty Lowland | Lake | 70.96800306 | -154.9707566 |
| Arctic Peaty Lowland | non-patterned Drained Thaw Lake Basin | 70.96789891 | -156.4810919 |
| Arctic Peaty Lowland | Drained slope | 70.96687383 | -157.2760512 |
| Arctic Peaty Lowland | Low-center polygon | 70.96592864 | -154.821988 |
| Arctic Peaty Lowland | Lake | 70.95694332 | -154.7553766 |
| Arctic Peaty Lowland | Lake | 70.95387486 | -155.2572127 |
| Arctic Peaty Lowland | Coalecent low-center polygon | 70.9527314 | -157.1619045 |
| Arctic Peaty Lowland | non-patterned Drained Thaw Lake Basin | 70.9499901 | -156.4785952 |
| Arctic Peaty Lowland | Lake | 70.94118138 | -154.6526839 |
| Arctic Peaty Lowland | High-center polygon | 70.93588723 | -157.464917 |
| Arctic Peaty Lowland | River | 70.90792458 | -156.1478621 |
| Arctic Peaty Lowland | River | 70.90781718 | -156.3558905 |
| Arctic Peaty Lowland | Low-center polygon | 70.90599812 | -156.9456423 |
| Arctic Peaty Lowland | Low-center polygon | 70.89950079 | -154.8610995 |
| Arctic Peaty Lowland | Lake | 70.89695677 | -157.1275882 |
| Arctic Peaty Lowland | Low-center polygon | 70.87694016 | -155.0020265 |
| Arctic Peaty Lowland | Low-center polygon | 70.87476248 | -156.4539106 |
| Arctic Peaty Lowland | Low-center polygon | 70.8742285 | -153.2097937 |
| Arctic Peaty Lowland | Low-center polygon | 70.87294327 | -153.5433008 |
| Arctic Peaty Lowland | Low-center polygon | 70.87252443 | -156.9008951 |
| Arctic Peaty Lowland | Flat-center polygon | 70.86936924 | -156.2974548 |
| Arctic Peaty Lowland | Coastal saline water | 70.86620875 | -152.8657335 |
| Arctic Peaty Lowland | High-center polygon | 70.86508951 | -153.1759423 |
| Arctic Peaty Lowland | Lake | 70.86408203 | -153.0158628 |
| Arctic Peaty Lowland | Low-center polygon | 70.86222466 | -155.1398777 |
| Arctic Peaty Lowland | Coastal saline water | 70.85712904 | -159.1898807 |
| Arctic Peaty Lowland | High-center polygon | 70.85424021 | -157.7808013 |
| Arctic Peaty Lowland | Low-center polygon | 70.85344693 | -152.9635474 |
| Arctic Peaty Lowland | non-patterned Drained Thaw Lake Basin | 70.85120704 | -153.7662314 |
| Arctic Peaty Lowland | non-patterned Drained Thaw Lake Basin | 70.84990071 | -153.6993365 |
| Arctic Peaty Lowland | Low-center polygon | 70.84911582 | -153.9196431 |
| Arctic Peaty Lowland | Low-center polygon | 70.84520065 | -156.8707774 |
| Arctic Peaty Lowland | High-center polygon | 70.83977088 | -156.8666798 |
| Arctic Peaty Lowland | River | 70.83793427 | -156.0410702 |
| Arctic Peaty Lowland | Low-center polygon | 70.83571133 | -156.4793156 |
| Arctic Peaty Lowland | Lake | 70.83150454 | -152.4438672 |
| Arctic Peaty Lowland | Lake | 70.83055604 | -155.2993886 |
| Arctic Peaty Lowland | Lake | 70.83017115 | -154.7731593 |
| Arctic Peaty Lowland | Coastal saline water | 70.82642795 | -158.8857527 |
| Arctic Peaty Lowland | Flat-center polygon | 70.82425486 | -159.2707106 |
| Arctic Peaty Lowland | non-patterned Drained Thaw Lake Basin | 70.82385845 | -156.9000756 |
| Arctic Peaty Lowland | non-patterned Drained Thaw Lake Basin | 70.821622 | -157.8770234 |
| Arctic Peaty Lowland | Low-center polygon | 70.8212284 | -156.1748221 |
| Arctic Peaty Lowland | Low-center polygon | 70.82057576 | -157.7891313 |
| Arctic Peaty Lowland | Flat-center polygon | 70.81980662 | -156.4934885 |
| Arctic Peaty Lowland | Coastal saline water | 70.81979698 | -158.825954 |
| Arctic Peaty Lowland | Low-center polygon | 70.81444533 | -152.7982522 |
| Arctic Peaty Lowland | Lake | 70.81376186 | -157.930596 |
| Arctic Peaty Lowland | Coastal saline water | 70.81176855 | -159.1710319 |
| Arctic Peaty Lowland | Coastal saline water | 70.80713888 | -158.9516218 |
| Arctic Peaty Lowland | High-center polygon | 70.80123592 | -157.9675415 |
| Arctic Peaty Lowland | Lake | 70.80059326 | -153.7875485 |
| Arctic Peaty Lowland | High-center polygon | 70.79939221 | -153.5411265 |
| Arctic Peaty Lowland | non-patterned Drained Thaw Lake Basin | 70.79791221 | -156.8100893 |
| Arctic Peaty Lowland | Low-center polygon | 70.79576548 | -156.0918829 |
| Arctic Peaty Lowland | Low-center polygon | 70.79338 | -156.6724248 |
| Arctic Peaty Lowland | Lake | 70.79210662 | -152.9656667 |
| Arctic Peaty Lowland | High-center polygon | 70.7904409 | -153.8802886 |
| Arctic Peaty Lowland | non-patterned Drained Thaw Lake Basin | 70.78838505 | -155.3707763 |
| Arctic Peaty Lowland | Coalecent low-center polygon | 70.78764968 | -155.8096717 |
| Arctic Peaty Lowland | Lake | 70.78523579 | -152.3789418 |
| Arctic Peaty Lowland | Low-center polygon | 70.78495794 | -157.2570844 |
| Arctic Peaty Lowland | Lake | 70.78405437 | -152.5690917 |
| Arctic Peaty Lowland | Lake | 70.78354143 | -153.4582914 |
| Arctic Peaty Lowland | Low-center polygon | 70.78303281 | -154.4124682 |
| Arctic Peaty Lowland | High-center polygon | 70.7802763 | -158.3025574 |
| Arctic Peaty Lowland | Low-center polygon | 70.78013073 | -152.3355934 |
| Arctic Peaty Lowland | Drained slope | 70.77882863 | -158.7387013 |
| Arctic Peaty Lowland | Lake | 70.77828209 | -156.8259982 |
| Arctic Peaty Lowland | non-patterned Drained Thaw Lake Basin | 70.77509509 | -156.2933681 |
| Arctic Peaty Lowland | High-center polygon | 70.77118712 | -156.3666919 |
| Arctic Peaty Lowland | Lake | 70.76931579 | -153.5443033 |
| Arctic Peaty Lowland | Lake | 70.76822869 | -156.6003102 |
| Arctic Peaty Lowland | Pond | 70.76735162 | -155.0919892 |
| Arctic Peaty Lowland | Lake | 70.76554223 | -156.9606821 |
| Arctic Peaty Lowland | Low-center polygon | 70.76497138 | -155.5144134 |
| Arctic Peaty Lowland | Drained slope | 70.76333955 | -158.7807208 |
| Arctic Peaty Lowland | Coastal saline water | 70.76238768 | -159.1761494 |
| Arctic Peaty Lowland | Lake | 70.76165243 | -155.6594029 |
| Arctic Peaty Lowland | Lake | 70.76040419 | -156.6029909 |
| Arctic Peaty Lowland | non-patterned Drained Thaw Lake Basin | 70.75867083 | -152.378464 |
| Arctic Peaty Lowland | Lake | 70.75703459 | -155.3393004 |
| Arctic Peaty Lowland | Low-center polygon | 70.75506429 | -152.4812709 |
| Arctic Peaty Lowland | Coalecent low-center polygon | 70.75387534 | -155.1961572 |
| Arctic Peaty Lowland | Sand dune | 70.74861542 | -154.4205784 |
| Arctic Peaty Lowland | High-center polygon | 70.74825356 | -156.5759076 |
| Arctic Peaty Lowland | Coalecent low-center polygon | 70.74282616 | -159.3461388 |
| Arctic Peaty Lowland | Lake | 70.7366176 | -158.0819055 |
| Arctic Peaty Lowland | Drained slope | 70.72835444 | -158.8276401 |
| Arctic Peaty Lowland | High-center polygon | 70.722649 | -155.972159 |
| Arctic Peaty Lowland | Lake | 70.71835347 | -156.0467835 |
| Arctic Peaty Lowland | Low-center polygon | 70.71747024 | -158.0094946 |
| Arctic Peaty Lowland | Lake | 70.71674516 | -154.0926539 |
| Arctic Peaty Lowland | Low-center polygon | 70.71656741 | -158.2281384 |
| Arctic Peaty Lowland | Lake | 70.71504301 | -153.7180612 |
| Arctic Peaty Lowland | Lake | 70.71331395 | -155.7102195 |
| Arctic Peaty Lowland | Lake | 70.71298392 | -156.0721983 |
| Arctic Peaty Lowland | Lake | 70.71198924 | -156.0890476 |
| Arctic Peaty Lowland | Lake | 70.70355439 | -156.7642947 |
| Arctic Peaty Lowland | High-center polygon | 70.70188162 | -159.7512216 |
| Arctic Peaty Lowland | non-patterned Drained Thaw Lake Basin | 70.70181248 | -156.1061783 |
| Arctic Peaty Lowland | Lake | 70.69901732 | -153.6268216 |
| Arctic Peaty Lowland | Low-center polygon | 70.69585179 | -153.3435316 |
| Arctic Peaty Lowland | Low-center polygon | 70.69436286 | -154.0626585 |
| Arctic Peaty Lowland | Pond | 70.69424335 | -157.1558846 |
| Arctic Peaty Lowland | High-center polygon | 70.69254533 | -156.8392663 |
| Arctic Peaty Lowland | Drained slope | 70.68971721 | -158.82155 |
| Arctic Peaty Lowland | High-center polygon | 70.6891253 | -158.9263922 |
| Arctic Peaty Lowland | Low-center polygon | 70.68763747 | -156.2219814 |
| Arctic Peaty Lowland | High-center polygon | 70.68681814 | -159.3446221 |
| Arctic Peaty Lowland | Lake | 70.68502258 | -153.7876798 |
| Arctic Peaty Lowland | Low-center polygon | 70.68360488 | -158.1409759 |
| Arctic Peaty Lowland | Low-center polygon | 70.68128148 | -155.2132333 |
| Arctic Peaty Lowland | High-center polygon | 70.67622709 | -159.2357793 |
| Arctic Peaty Lowland | High-center polygon | 70.67426253 | -159.3135372 |
| Arctic Peaty Lowland | non-patterned Drained Thaw Lake Basin | 70.67350645 | -159.7895733 |
| Arctic Peaty Lowland | non-patterned Drained Thaw Lake Basin | 70.67328796 | -157.7455322 |
| Arctic Peaty Lowland | Low-center polygon | 70.67294238 | -152.7533981 |
| Arctic Peaty Lowland | High-center polygon | 70.67202678 | -159.9101875 |
| Arctic Peaty Lowland | Lake | 70.66937882 | -155.5421445 |
| Arctic Peaty Lowland | Drained slope | 70.66343156 | -159.4111716 |
| Arctic Peaty Lowland | Low-center polygon | 70.6633801 | -157.8962996 |
| Arctic Peaty Lowland | Low-center polygon | 70.65905487 | -157.3706246 |
| Arctic Peaty Lowland | Coalecent low-center polygon | 70.65674393 | -154.0938066 |
| Arctic Peaty Lowland | Lake | 70.65594756 | -155.629001 |
| Arctic Peaty Lowland | non-patterned Drained Thaw Lake Basin | 70.65307288 | -155.1325497 |
| Arctic Peaty Lowland | non-patterned Drained Thaw Lake Basin | 70.65302162 | -152.9677224 |
| Arctic Peaty Lowland | Lake | 70.6526144 | -156.7007731 |
| Arctic Peaty Lowland | High-center polygon | 70.64737421 | -156.3494994 |
| Arctic Peaty Lowland | Coalecent low-center polygon | 70.64468162 | -159.1440184 |
| Arctic Peaty Lowland | Lake | 70.64148534 | -153.3179802 |
| Arctic Peaty Lowland | High-center polygon | 70.64045297 | -154.4553041 |
| Arctic Peaty Lowland | High-center polygon | 70.63497507 | -159.5822955 |
| Arctic Peaty Lowland | Lake | 70.63219875 | -153.8485992 |
| Arctic Peaty Lowland | High-center polygon | 70.63034325 | -159.2115764 |
| Arctic Peaty Lowland | Drained slope | 70.62693603 | -152.751355 |
| Arctic Peaty Lowland | Low-center polygon | 70.62556351 | -152.6179081 |
| Arctic Peaty Lowland | Lake | 70.62501683 | -153.151979 |
| Arctic Peaty Lowland | Low-center polygon | 70.62187754 | -158.2916408 |
| Arctic Peaty Lowland | Flat-center polygon | 70.61518361 | -155.0775433 |
| Arctic Peaty Lowland | Lake | 70.6137052 | -156.5004187 |
| Arctic Peaty Lowland | Low-center polygon | 70.61291656 | -155.8753322 |
| Arctic Peaty Lowland | Lake | 70.60834587 | -153.259528 |
| Arctic Peaty Lowland | Lake | 70.60558052 | -159.4532831 |
| Arctic Peaty Lowland | non-patterned Drained Thaw Lake Basin | 70.60134414 | -159.6364388 |
| Arctic Peaty Lowland | Coalecent low-center polygon | 70.60044547 | -156.3399724 |
| Arctic Peaty Lowland | Lake | 70.59701581 | -155.2300394 |
| Arctic Peaty Lowland | Lake | 70.59602006 | -153.3113584 |
| Arctic Peaty Lowland | non-patterned Drained Thaw Lake Basin | 70.59234256 | -154.3435573 |
| Arctic Peaty Lowland | Low-center polygon | 70.58996553 | -154.367428 |
| Arctic Peaty Lowland | Lake | 70.58867938 | -159.2894068 |
| Arctic Peaty Lowland | non-patterned Drained Thaw Lake Basin | 70.58310109 | -156.4343685 |
| Arctic Peaty Lowland | Lake | 70.5812057 | -153.298337 |
| Arctic Peaty Lowland | High-center polygon | 70.58101564 | -152.5971627 |
| Arctic Peaty Lowland | Lake | 70.58100739 | -153.1025327 |
| Arctic Peaty Lowland | Low-center polygon | 70.57907937 | -159.5010215 |
| Arctic Peaty Lowland | High-center polygon | 70.57793189 | -157.8072276 |
| Arctic Peaty Lowland | Coalecent low-center polygon | 70.57705488 | -155.0150997 |
| Arctic Peaty Lowland | non-patterned Drained Thaw Lake Basin | 70.57676851 | -156.192578 |
| Arctic Peaty Lowland | non-patterned Drained Thaw Lake Basin | 70.5765798 | -156.3991627 |
| Arctic Peaty Lowland | Low-center polygon | 70.57512632 | -154.9610445 |
| Arctic Peaty Lowland | High-center polygon | 70.57173035 | -160.0288253 |
| Arctic Peaty Lowland | Lake | 70.57014988 | -158.7224242 |
| Arctic Peaty Lowland | Lake | 70.56713757 | -158.7423278 |
| Arctic Peaty Lowland | Low-center polygon | 70.5644181 | -154.1594488 |
| Arctic Peaty Lowland | Lake | 70.56428336 | -153.3254687 |
| Arctic Peaty Lowland | Lake | 70.56366838 | -156.0908564 |
| Arctic Peaty Lowland | High-center polygon | 70.56048178 | -158.8866112 |
| Arctic Peaty Lowland | Low-center polygon | 70.55912295 | -151.9780734 |
| Arctic Peaty Lowland | Lake | 70.55861918 | -153.9582144 |
| Arctic Peaty Lowland | Lake | 70.55334327 | -154.9863258 |
| Arctic Peaty Lowland | Coalecent low-center polygon | 70.54733606 | -152.6963919 |
| Arctic Peaty Lowland | non-patterned Drained Thaw Lake Basin | 70.54609061 | -158.9578002 |
| Arctic Peaty Lowland | Low-center polygon | 70.54518514 | -154.4889647 |
| Arctic Peaty Lowland | Lake | 70.54259966 | -156.7772054 |
| Arctic Peaty Lowland | Lake | 70.54172169 | -154.0013214 |
| Arctic Peaty Lowland | Flat-center polygon | 70.54102539 | -154.8423759 |
| Arctic Peaty Lowland | Drained slope | 70.53640717 | -152.1331461 |
| Arctic Peaty Lowland | non-patterned Drained Thaw Lake Basin | 70.53626741 | -157.8874704 |
| Arctic Peaty Lowland | Drained slope | 70.53434151 | -157.581606 |
| Arctic Peaty Lowland | Lake | 70.53396019 | -155.7322309 |
| Arctic Peaty Lowland | Lake | 70.53261908 | -157.915215 |
| Arctic Peaty Lowland | High-center polygon | 70.53155679 | -159.5912772 |
| Arctic Peaty Lowland | High-center polygon | 70.52912653 | -160.2685885 |
| Arctic Peaty Lowland | Lake | 70.52627175 | -153.3092888 |
| Arctic Peaty Lowland | Lake | 70.52605858 | -153.7598072 |
| Arctic Peaty Lowland | Flat-center polygon | 70.52212397 | -158.454692 |
| Arctic Peaty Lowland | Low-center polygon | 70.51977626 | -153.0350717 |
| Arctic Peaty Lowland | High-center polygon | 70.51965105 | -159.48897 |
| Arctic Peaty Lowland | Low-center polygon | 70.51702845 | -154.2132873 |
| Arctic Peaty Lowland | High-center polygon | 70.51205365 | -152.2608026 |
| Arctic Peaty Lowland | Lake | 70.50950742 | -158.3088926 |
| Arctic Peaty Lowland | Coalecent low-center polygon | 70.50930325 | -156.5591007 |
| Arctic Peaty Lowland | Lake | 70.50830807 | -156.1416482 |
| Arctic Peaty Lowland | Low-center polygon | 70.507766 | -152.2903419 |
| Arctic Peaty Lowland | non-patterned Drained Thaw Lake Basin | 70.50687297 | -152.7932114 |
| Arctic Peaty Lowland | non-patterned Drained Thaw Lake Basin | 70.50573138 | -156.1881428 |
| Arctic Peaty Lowland | High-center polygon | 70.50470821 | -153.0597405 |
| Arctic Peaty Lowland | Low-center polygon | 70.50341248 | -154.206163 |
| Arctic Peaty Lowland | Lake | 70.49892312 | -154.1798342 |
| Arctic Peaty Lowland | Low-center polygon | 70.49774927 | -152.8110858 |
| Arctic Peaty Lowland | High-center polygon | 70.49181882 | -159.647182 |
| Arctic Peaty Lowland | Low-center polygon | 70.4915343 | -158.286441 |
| Arctic Peaty Lowland | Low-center polygon | 70.49127119 | -158.3494212 |
| Arctic Peaty Lowland | High-center polygon | 70.48669248 | -154.5526206 |
| Arctic Peaty Lowland | High-center polygon | 70.48563332 | -151.9779983 |
| Arctic Peaty Lowland | High-center polygon | 70.484665 | -159.4936256 |
| Arctic Peaty Lowland | Coastal saline water | 70.48438144 | -159.9244085 |
| Arctic Peaty Lowland | Lake | 70.48434864 | -153.8318113 |
| Arctic Peaty Lowland | Low-center polygon | 70.48240446 | -158.5270722 |
| Arctic Peaty Lowland | Coastal saline water | 70.48109525 | -159.8183087 |
| Arctic Peaty Lowland | Lake | 70.47988716 | -153.8887198 |
| Arctic Peaty Lowland | Lake | 70.47803888 | -152.4428468 |
| Arctic Peaty Lowland | Low-center polygon | 70.47103563 | -154.2925411 |
| Arctic Peaty Lowland | High-center polygon | 70.47050349 | -159.1196176 |
| Arctic Peaty Lowland | Low-center polygon | 70.47023544 | -154.539978 |
| Arctic Peaty Lowland | Low-center polygon | 70.47007366 | -158.7702986 |
| Arctic Peaty Lowland | Coastal saline water | 70.46944307 | -159.8306627 |
| Arctic Peaty Lowland | Coastal saline water | 70.46834356 | -160.0585342 |
| Arctic Peaty Lowland | Coalecent low-center polygon | 70.45711315 | -150.7444308 |
| Arctic Peaty Lowland | Lake | 70.45669512 | -155.8605307 |
| Arctic Peaty Lowland | Coalecent low-center polygon | 70.4525049 | -149.4783612 |
| Arctic Peaty Lowland | Lake | 70.45202971 | -160.0337234 |
| Arctic Peaty Lowland | Drained slope | 70.44936771 | -160.2096258 |
| Arctic Peaty Lowland | High-center polygon | 70.44720569 | -152.1021561 |
| Arctic Peaty Lowland | Pond | 70.44331341 | -149.8819514 |
| Arctic Peaty Lowland | Lake | 70.4430442 | -152.2305046 |
| Arctic Peaty Lowland | Lake | 70.43632695 | -152.0701033 |
| Arctic Peaty Lowland | High-center polygon | 70.43428938 | -159.1787082 |
| Arctic Peaty Lowland | Lake | 70.43384462 | -152.5474206 |
| Arctic Peaty Lowland | Low-center polygon | 70.42672572 | -158.9440983 |
| Arctic Peaty Lowland | Coastal saline water | 70.42578844 | -151.1750354 |
| Arctic Peaty Lowland | Low-center polygon | 70.42390645 | -158.5432497 |
| Arctic Peaty Lowland | High-center polygon | 70.41964064 | -159.4905158 |
| Arctic Peaty Lowland | Sandy barren | 70.41955943 | -155.8252216 |
| Arctic Peaty Lowland | Pond | 70.4184751 | -159.7977112 |
| Arctic Peaty Lowland | River | 70.41458045 | -148.8877477 |
| Arctic Peaty Lowland | River | 70.41177606 | -148.8886988 |
| Arctic Peaty Lowland | Low-center polygon | 70.40953533 | -149.4518084 |
| Arctic Peaty Lowland | High-center polygon | 70.40769001 | -160.4050262 |
| Arctic Peaty Lowland | Drained slope | 70.40635545 | -160.6218368 |
| Arctic Peaty Lowland | Coastal saline water | 70.40625476 | -159.8857926 |
| Arctic Peaty Lowland | High-center polygon | 70.39929836 | -159.8287967 |
| Arctic Peaty Lowland | non-patterned Drained Thaw Lake Basin | 70.39900719 | -158.4739352 |
| Arctic Peaty Lowland | Lake | 70.3983793 | -160.0997634 |
| Arctic Peaty Lowland | Lake | 70.39556541 | -151.0618341 |
| Arctic Peaty Lowland | Lake | 70.39549601 | -149.6380022 |
| Arctic Peaty Lowland | Drained slope | 70.38719409 | -148.8142322 |
| Arctic Peaty Lowland | Coastal saline water | 70.38620376 | -159.9837051 |
| Arctic Peaty Lowland | High-center polygon | 70.38564128 | -150.1036602 |
| Arctic Peaty Lowland | Low-center polygon | 70.37550446 | -158.9186106 |
| Arctic Peaty Lowland | Coastal saline water | 70.37527365 | -148.5188196 |
| Arctic Peaty Lowland | High-center polygon | 70.37494568 | -151.4013502 |
| Arctic Peaty Lowland | Lake | 70.37288994 | -154.6198359 |
| Arctic Peaty Lowland | High-center polygon | 70.37225074 | -157.0304521 |
| Arctic Peaty Lowland | Coastal saline water | 70.37198307 | -159.9558494 |
| Arctic Peaty Lowland | Coastal saline water | 70.37182267 | -159.9763022 |
| Arctic Peaty Lowland | Low-center polygon | 70.36993964 | -151.3726043 |
| Arctic Peaty Lowland | High-center polygon | 70.36735011 | -158.6257629 |
| Arctic Peaty Lowland | Drained slope | 70.36618931 | -159.417132 |
| Arctic Peaty Lowland | Low-center polygon | 70.36548479 | -150.8424541 |
| Arctic Peaty Lowland | Drained slope | 70.36404917 | -160.1469401 |
| Arctic Peaty Lowland | Low-center polygon | 70.35818072 | -149.3264524 |
| Arctic Peaty Lowland | High-center polygon | 70.35793952 | -158.8005861 |
| Arctic Peaty Lowland | Low-center polygon | 70.35357077 | -150.799419 |
| Arctic Peaty Lowland | Low-center polygon | 70.35213535 | -153.2132208 |
| Arctic Peaty Lowland | Drained slope | 70.35111087 | -149.535972 |
| Arctic Peaty Lowland | Coalecent low-center polygon | 70.34684977 | -159.601822 |
| Arctic Peaty Lowland | High-center polygon | 70.34639615 | -149.6858496 |
| Arctic Peaty Lowland | Lake | 70.34627256 | -151.4126646 |
| Arctic Peaty Lowland | High-center polygon | 70.34512793 | -156.9733652 |
| Arctic Peaty Lowland | High-center polygon | 70.34399013 | -160.1770092 |
| Arctic Peaty Lowland | Coastal saline water | 70.33975611 | -160.1639407 |
| Arctic Peaty Lowland | Sandy barren | 70.33964717 | -153.3100684 |
| Arctic Peaty Lowland | High-center polygon | 70.33599371 | -158.1225528 |
| Arctic Peaty Lowland | Low-center polygon | 70.33431139 | -150.8245587 |
| Arctic Peaty Lowland | Drained slope | 70.33382117 | -150.0682693 |
| Arctic Peaty Lowland | Lake | 70.33224869 | -149.1254187 |
| Arctic Peaty Lowland | Low-center polygon | 70.33010021 | -159.2616974 |
| Arctic Peaty Lowland | Low-center polygon | 70.329904 | -150.6043216 |
| Arctic Peaty Lowland | Flat-center polygon | 70.3275672 | -150.2636362 |
| Arctic Peaty Lowland | Lake | 70.32749717 | -149.9571025 |
| Arctic Peaty Lowland | Low-center polygon | 70.31929177 | -160.4317129 |
| Arctic Peaty Lowland | Flat-center polygon | 70.31905755 | -149.5776617 |
| Arctic Peaty Lowland | Drained slope | 70.31819719 | -150.376838 |
| Arctic Peaty Lowland | Low-center polygon | 70.31408911 | -153.421266 |
| Arctic Peaty Lowland | Low-center polygon | 70.31342454 | -151.0954806 |
| Arctic Peaty Lowland | Low-center polygon | 70.31207623 | -157.7797083 |
| Arctic Peaty Lowland | Drained slope | 70.30706414 | -158.5676761 |
| Arctic Peaty Lowland | Lake | 70.30625195 | -149.132697 |
| Arctic Peaty Lowland | High-center polygon | 70.30577875 | -160.7681272 |
| Arctic Peaty Lowland | Sandy barren | 70.30260233 | -161.9565424 |
| Arctic Peaty Lowland | River | 70.29898109 | -148.100245 |
| Arctic Peaty Lowland | Low-center polygon | 70.29859439 | -150.4159569 |
| Arctic Peaty Lowland | Sandy barren | 70.29770948 | -161.7770979 |
| Arctic Peaty Lowland | Low-center polygon | 70.296031 | -156.876999 |
| Arctic Peaty Lowland | Coastal saline water | 70.29526915 | -161.2613022 |
| Arctic Peaty Lowland | High-center polygon | 70.29374965 | -160.1383513 |
| Arctic Peaty Lowland | High-center polygon | 70.28805661 | -159.5242639 |
| Arctic Peaty Lowland | High-center polygon | 70.28421946 | -148.7574462 |
| Arctic Peaty Lowland | High-center polygon | 70.2826518 | -158.8931379 |
| Arctic Peaty Lowland | Lake | 70.28235207 | -150.1087098 |
| Arctic Peaty Lowland | High-center polygon | 70.28221326 | -161.1014675 |
| Arctic Peaty Lowland | Sand dune | 70.2817602 | -148.2491302 |
| Arctic Peaty Lowland | High-center polygon | 70.28126158 | -149.7059796 |
| Arctic Peaty Lowland | Coastal saline water | 70.27746325 | -161.6145268 |
| Arctic Peaty Lowland | Lake | 70.27721594 | -158.4251829 |
| Arctic Peaty Lowland | Flat-center polygon | 70.27363246 | -148.3437588 |
| Arctic Peaty Lowland | Lake | 70.27351439 | -148.7748574 |
| Arctic Peaty Lowland | Sandy barren | 70.2734407 | -147.8763449 |
| Arctic Peaty Lowland | Lake | 70.27217533 | -159.1204704 |
| Arctic Peaty Lowland | non-patterned Drained Thaw Lake Basin | 70.27212032 | -160.7370912 |
| Arctic Peaty Lowland | High-center polygon | 70.27090262 | -151.4681357 |
| Arctic Peaty Lowland | Low-center polygon | 70.26870764 | -156.8147206 |
| Arctic Peaty Lowland | Flat-center polygon | 70.26525724 | -160.7467461 |
| Arctic Peaty Lowland | Low-center polygon | 70.26294534 | -160.7029705 |
| Arctic Peaty Lowland | Low-center polygon | 70.26208912 | -149.4964919 |
| Arctic Peaty Lowland | High-center polygon | 70.25343279 | -148.4960737 |
| Arctic Peaty Lowland | Lake | 70.25097882 | -158.0062802 |
| Arctic Peaty Lowland | Drained slope | 70.24836588 | -159.8294545 |
| Arctic Peaty Lowland | Low-center polygon | 70.24628646 | -149.3084891 |
| Arctic Peaty Lowland | Low-center polygon | 70.24468657 | -160.5838679 |
| Arctic Peaty Lowland | Flat-center polygon | 70.24205207 | -151.0166488 |
| Arctic Peaty Lowland | Pond | 70.2399468 | -159.4957172 |
| Arctic Peaty Lowland | Coalecent low-center polygon | 70.2397169 | -148.922416 |
| Arctic Peaty Lowland | Low-center polygon | 70.23951723 | -161.9728909 |
| Arctic Peaty Lowland | Coastal saline water | 70.23765139 | -162.1611158 |
| Arctic Peaty Lowland | High-center polygon | 70.23610684 | -151.2818593 |
| Arctic Peaty Lowland | Sand dune | 70.2356893 | -149.0071104 |
| Arctic Peaty Lowland | High-center polygon | 70.23472868 | -158.9819584 |
| Arctic Peaty Lowland | Lake | 70.23450776 | -161.2114491 |
| Arctic Peaty Lowland | High-center polygon | 70.23308277 | -160.183982 |
| Arctic Peaty Lowland | Lake | 70.22718341 | -151.5831468 |
| Arctic Peaty Lowland | Lake | 70.22629423 | -151.5339822 |
| Arctic Peaty Lowland | High-center polygon | 70.22557557 | -160.2841592 |
| Arctic Peaty Lowland | Coastal saline water | 70.22514187 | -161.7384533 |
| Arctic Peaty Lowland | Coastal saline water | 70.22478702 | -161.769068 |
| Arctic Peaty Lowland | Low-center polygon | 70.22088067 | -151.6325199 |
| Arctic Peaty Lowland | High-center polygon | 70.21995927 | -160.0987497 |
| Arctic Peaty Lowland | non-patterned Drained Thaw Lake Basin | 70.21923662 | -154.9456158 |
| Arctic Peaty Lowland | Flat-center polygon | 70.21878515 | -148.1211891 |
| Arctic Peaty Lowland | Drained slope | 70.2164393 | -151.955118 |
| Arctic Peaty Lowland | High-center polygon | 70.21616559 | -160.1885686 |
| Arctic Peaty Lowland | Sandy barren | 70.21490186 | -148.289994 |
| Arctic Peaty Lowland | non-patterned Drained Thaw Lake Basin | 70.2131563 | -151.9765193 |
| Arctic Peaty Lowland | High-center polygon | 70.21254074 | -151.5538181 |
| Arctic Peaty Lowland | Drained slope | 70.21222092 | -160.3950859 |
| Arctic Peaty Lowland | Drained slope | 70.20952517 | -160.4038836 |
| Arctic Peaty Lowland | Coastal saline water | 70.20804593 | -161.7479034 |
| Arctic Peaty Lowland | Drained slope | 70.20658959 | -158.9437856 |
| Arctic Peaty Lowland | High-center polygon | 70.20639115 | -149.6012421 |
| Arctic Peaty Lowland | non-patterned Drained Thaw Lake Basin | 70.20519904 | -151.6833903 |
| Arctic Peaty Lowland | High-center polygon | 70.20282227 | -151.0289146 |
| Arctic Peaty Lowland | Low-center polygon | 70.2020254 | -150.8028859 |
| Arctic Peaty Lowland | Lake | 70.19916435 | -161.327594 |
| Arctic Peaty Lowland | non-patterned Drained Thaw Lake Basin | 70.1967596 | -153.137849 |
| Arctic Peaty Lowland | Drained slope | 70.1961716 | -158.9162258 |
| Arctic Peaty Lowland | High-center polygon | 70.19606328 | -152.0940208 |
| Arctic Peaty Lowland | non-patterned Drained Thaw Lake Basin | 70.19558738 | -154.7528797 |
| Arctic Peaty Lowland | Low-center polygon | 70.19446765 | -147.6270372 |
| Arctic Peaty Lowland | High-center polygon | 70.19106829 | -162.0545435 |
| Arctic Peaty Lowland | non-patterned Drained Thaw Lake Basin | 70.19099183 | -150.6228172 |
| Arctic Peaty Lowland | Flat-center polygon | 70.18955392 | -161.9741552 |
| Arctic Peaty Lowland | Coastal saline water | 70.18905509 | -147.37193 |
| Arctic Peaty Lowland | River | 70.18754944 | -149.0440813 |
| Arctic Peaty Lowland | non-patterned Drained Thaw Lake Basin | 70.18672743 | -162.069753 |
| Arctic Peaty Lowland | Low-center polygon | 70.18653448 | -157.8997074 |
| Arctic Peaty Lowland | Lake | 70.1850906 | -159.4541817 |
| Arctic Peaty Lowland | High-center polygon | 70.18297848 | -147.9292902 |
| Arctic Peaty Lowland | Low-center polygon | 70.18115947 | -162.0377482 |
| Arctic Peaty Lowland | River | 70.18108504 | -147.2617363 |
| Arctic Peaty Lowland | Low-center polygon | 70.1808856 | -160.0781795 |
| Arctic Peaty Lowland | High-center polygon | 70.17995416 | -148.6147237 |
| Arctic Peaty Lowland | High-center polygon | 70.17888049 | -148.6510917 |
| Arctic Peaty Lowland | Drained slope | 70.17643688 | -157.6576373 |
| Arctic Peaty Lowland | High-center polygon | 70.17564008 | -161.4291749 |
| Arctic Peaty Lowland | Flat-center polygon | 70.17544096 | -147.4021693 |
| Arctic Peaty Lowland | Lake | 70.1748174 | -155.9901542 |
| Arctic Peaty Lowland | High-center polygon | 70.17209664 | -146.3370918 |
| Arctic Peaty Lowland | Pond | 70.17047561 | -147.7183098 |
| Arctic Peaty Lowland | High-center polygon | 70.16959687 | -151.6061922 |
| Arctic Peaty Lowland | High-center polygon | 70.16720374 | -160.7044143 |
| Arctic Peaty Lowland | Sand dune | 70.16688459 | -156.6029005 |
| Arctic Peaty Lowland | High-center polygon | 70.1667438 | -161.3213301 |
| Arctic Peaty Lowland | High-center polygon | 70.16596497 | -162.0439084 |
| Arctic Peaty Lowland | Riparian corridor | 70.16545585 | -148.4265964 |
| Arctic Peaty Lowland | Low-center polygon | 70.15970184 | -158.1747014 |
| Arctic Peaty Lowland | Flat-center polygon | 70.15550915 | -148.9298648 |
| Arctic Peaty Lowland | High-center polygon | 70.1553863 | -162.1217103 |
| Arctic Peaty Lowland | Low-center polygon | 70.15466865 | -160.7730217 |
| Arctic Peaty Lowland | Flat-center polygon | 70.15082112 | -148.2314781 |
| Arctic Peaty Lowland | High-center polygon | 70.15049758 | -161.8695643 |
| Arctic Peaty Lowland | High-center polygon | 70.14895964 | -161.0169271 |
| Arctic Peaty Lowland | Drained slope | 70.14878483 | -160.1837127 |
| Arctic Peaty Lowland | non-patterned Drained Thaw Lake Basin | 70.14709492 | -153.3900011 |
| Arctic Peaty Lowland | Coalecent low-center polygon | 70.14663449 | -147.7988305 |
| Arctic Peaty Lowland | High-center polygon | 70.14646867 | -146.8616299 |
| Arctic Peaty Lowland | River | 70.14511678 | -145.9033029 |
| Arctic Peaty Lowland | Low-center polygon | 70.14401179 | -147.764208 |
| Arctic Peaty Lowland | High-center polygon | 70.14335787 | -158.4337839 |
| Arctic Peaty Lowland | Drained slope | 70.14255294 | -158.5045516 |
| Arctic Peaty Lowland | High-center polygon | 70.14217015 | -149.2636521 |
| Arctic Peaty Lowland | High-center polygon | 70.1420049 | -151.4491816 |
| Arctic Peaty Lowland | High-center polygon | 70.14182017 | -158.7912999 |
| Arctic Peaty Lowland | High-center polygon | 70.14117972 | -161.8740286 |
| Arctic Peaty Lowland | Pond | 70.14103973 | -148.7875935 |
| Arctic Peaty Lowland | High-center polygon | 70.13725977 | -159.0573323 |
| Arctic Peaty Lowland | Drained slope | 70.13489511 | -160.1853108 |
| Arctic Peaty Lowland | High-center polygon | 70.1340124 | -159.9344503 |
| Arctic Peaty Lowland | Low-center polygon | 70.13275446 | -158.7620779 |
| Arctic Peaty Lowland | Drained slope | 70.1307126 | -150.7669842 |
| Arctic Peaty Lowland | High-center polygon | 70.12819925 | -147.8801348 |
| Arctic Peaty Lowland | Sandy barren | 70.12234015 | -152.9220178 |
| Arctic Peaty Lowland | Drained slope | 70.12199725 | -161.4742828 |
| Arctic Peaty Lowland | High-center polygon | 70.12075955 | -157.5526553 |
| Arctic Peaty Lowland | High-center polygon | 70.11991744 | -158.777901 |
| Arctic Peaty Lowland | Drained slope | 70.11909565 | -159.9358899 |
| Arctic Peaty Lowland | Low-center polygon | 70.11192665 | -158.4394768 |
| Arctic Peaty Lowland | High-center polygon | 70.11127732 | -157.9234348 |
| Arctic Peaty Lowland | Drained slope | 70.11027168 | -158.4833359 |
| Arctic Peaty Lowland | Drained slope | 70.1101686 | -158.9392839 |
| Arctic Peaty Lowland | High-center polygon | 70.10969203 | -149.4312609 |
| Arctic Peaty Lowland | Pond | 70.1090281 | -147.4210316 |
| Arctic Peaty Lowland | Low-center polygon | 70.10880805 | -148.7471075 |
| Arctic Peaty Lowland | Drained slope | 70.10665904 | -150.4847055 |
| Arctic Peaty Lowland | Drained slope | 70.10564899 | -149.1036907 |
| Arctic Peaty Lowland | Low-center polygon | 70.10534211 | -158.8007909 |
| Arctic Peaty Lowland | High-center polygon | 70.10262005 | -147.4317846 |
| Arctic Peaty Lowland | Riparian corridor | 70.10229626 | -148.5068869 |
| Arctic Peaty Lowland | Sand dune | 70.10047699 | -148.4663973 |
| Arctic Peaty Lowland | Low-center polygon | 70.09884514 | -149.0010533 |
| Arctic Peaty Lowland | Sand dune | 70.09575845 | -148.456002 |
| Arctic Peaty Lowland | Low-center polygon | 70.09571096 | -158.9297404 |
| Arctic Peaty Lowland | non-patterned Drained Thaw Lake Basin | 70.09488954 | -162.0939676 |
| Arctic Peaty Lowland | Coalecent low-center polygon | 70.09435863 | -152.2412902 |
| Arctic Peaty Lowland | High-center polygon | 70.09424657 | -146.3468302 |
| Arctic Peaty Lowland | Sandy barren | 70.093652 | -162.3855905 |
| Arctic Peaty Lowland | High-center polygon | 70.09287247 | -157.6095418 |
| Arctic Peaty Lowland | High-center polygon | 70.09249848 | -149.0525407 |
| Arctic Peaty Lowland | Flat-center polygon | 70.09214687 | -154.5957614 |
| Arctic Peaty Lowland | Low-center polygon | 70.08974707 | -147.768963 |
| Arctic Peaty Lowland | High-center polygon | 70.08936656 | -143.1902648 |
| Arctic Peaty Lowland | Low-center polygon | 70.08883531 | -160.0262874 |
| Arctic Peaty Lowland | High-center polygon | 70.08772346 | -143.1347157 |
| Arctic Peaty Lowland | High-center polygon | 70.08536981 | -150.9160433 |
| Arctic Peaty Lowland | Lake | 70.08273757 | -147.3875043 |
| Arctic Peaty Lowland | Low-center polygon | 70.08200061 | -160.0395275 |
| Arctic Peaty Lowland | Lake | 70.07975924 | -161.8924636 |
| Arctic Peaty Lowland | Low-center polygon | 70.07810959 | -143.2492366 |
| Arctic Peaty Lowland | Flat-center polygon | 70.07690417 | -143.2309335 |
| Arctic Peaty Lowland | Lake | 70.07639585 | -145.6555434 |
| Arctic Peaty Lowland | Low-center polygon | 70.07373864 | -157.4602027 |
| Arctic Peaty Lowland | Drained slope | 70.07363843 | -147.8832872 |
| Arctic Peaty Lowland | High-center polygon | 70.07070739 | -156.0590965 |
| Arctic Peaty Lowland | Pond | 70.06975639 | -148.2166821 |
| Arctic Peaty Lowland | Drained slope | 70.06930973 | -160.7229213 |
| Arctic Peaty Lowland | High-center polygon | 70.0680265 | -143.8541841 |
| Arctic Peaty Lowland | High-center polygon | 70.06536337 | -146.38014 |
| Arctic Peaty Lowland | Drained slope | 70.06183261 | -149.3966144 |
| Arctic Peaty Lowland | High-center polygon | 70.06135294 | -157.5168923 |
| Arctic Peaty Lowland | Low-center polygon | 70.06084783 | -157.2456043 |
| Arctic Peaty Lowland | Low-center polygon | 70.06055776 | -146.962716 |
| Arctic Peaty Lowland | Sand dune | 70.05843897 | -145.6978316 |
| Arctic Peaty Lowland | High-center polygon | 70.0565318 | -145.4881856 |
| Arctic Peaty Lowland | High-center polygon | 70.05606964 | -143.5270089 |
| Arctic Peaty Lowland | River | 70.05471398 | -144.1075546 |
| Arctic Peaty Lowland | High-center polygon | 70.05246969 | -151.8444216 |
| Arctic Peaty Lowland | Sandy barren | 70.05208781 | -151.1435742 |
| Arctic Peaty Lowland | High-center polygon | 70.05205192 | -161.9328927 |
| Arctic Peaty Lowland | High-center polygon | 70.05177476 | -143.4368452 |
| Arctic Peaty Lowland | High-center polygon | 70.05126174 | -161.8933205 |
| Arctic Peaty Lowland | Sandy barren | 70.05019295 | -162.4321382 |
| Arctic Peaty Lowland | High-center polygon | 70.04956148 | -157.3045073 |
| Arctic Peaty Lowland | Drained slope | 70.04886457 | -147.6437126 |
| Arctic Peaty Lowland | Sand dune | 70.04750044 | -154.6178447 |
| Arctic Peaty Lowland | High-center polygon | 70.04436246 | -143.0223331 |
| Arctic Peaty Lowland | Low-center polygon | 70.04427355 | -149.4609054 |
| Arctic Peaty Lowland | High-center polygon | 70.04343132 | -160.140454 |
| Arctic Peaty Lowland | Low-center polygon | 70.04060852 | -149.2832417 |
| Arctic Peaty Lowland | High-center polygon | 70.03931132 | -145.8213921 |
| Arctic Peaty Lowland | High-center polygon | 70.03646768 | -152.2002618 |
| Arctic Peaty Lowland | High-center polygon | 70.03457665 | -148.8266718 |
| Arctic Peaty Lowland | Low-center polygon | 70.0317229 | -160.525608 |
| Arctic Peaty Lowland | Low-center polygon | 70.02620531 | -151.1165609 |
| Arctic Peaty Lowland | Drained slope | 70.02493208 | -150.9819581 |
| Arctic Peaty Lowland | Coastal saline water | 70.02296029 | -162.5833285 |
| Arctic Peaty Lowland | Flat-center polygon | 70.01796704 | -147.8009572 |
| Arctic Peaty Lowland | High-center polygon | 70.01706573 | -143.1783318 |
| Arctic Peaty Lowland | Sand dune | 70.01663328 | -152.3628905 |
| Arctic Peaty Lowland | High-center polygon | 70.01381849 | -151.1711638 |
| Arctic Peaty Lowland | Flat-center polygon | 70.01055657 | -147.6176133 |
| Arctic Peaty Lowland | Low-center polygon | 70.01034997 | -153.1904493 |
| Arctic Peaty Lowland | High-center polygon | 70.0099623 | -147.6194573 |
| Arctic Peaty Lowland | High-center polygon | 70.00641792 | -147.6438107 |
| Arctic Peaty Lowland | High-center polygon | 70.00627501 | -146.5694678 |
| Arctic Peaty Lowland | Sandy barren | 70.00546395 | -151.6026127 |
| Arctic Peaty Lowland | Flat-center polygon | 70.00370706 | -144.0548079 |
| Arctic Peaty Lowland | Lake | 70.00053198 | -145.3406582 |
| Arctic Peaty Lowland | Low-center polygon | 69.99802578 | -145.5199388 |
| Arctic Peaty Lowland | Drained slope | 69.99742881 | -143.4057387 |
| Arctic Peaty Lowland | High-center polygon | 69.99660555 | -146.7317723 |
| Arctic Peaty Lowland | Sand dune | 69.99329737 | -154.5941464 |
| Arctic Peaty Lowland | High-center polygon | 69.99090248 | -148.7101839 |
| Arctic Peaty Lowland | High-center polygon | 69.99044148 | -143.9741823 |
| Arctic Peaty Lowland | High-center polygon | 69.9891683 | -146.3893779 |
| Arctic Peaty Lowland | Low-center polygon | 69.98634479 | -143.2847672 |
| Arctic Peaty Lowland | Drained slope | 69.98500932 | -147.377691 |
| Arctic Peaty Lowland | Sand dune | 69.98418739 | -155.3455009 |
| Arctic Peaty Lowland | High-center polygon | 69.98406105 | -147.7959227 |
| Arctic Peaty Lowland | Riparian corridor | 69.98204761 | -152.4858731 |
| Arctic Peaty Lowland | High-center polygon | 69.9819327 | -143.3811092 |
| Arctic Peaty Lowland | High-center polygon | 69.98006659 | -148.8689953 |
| Arctic Peaty Lowland | Low-center polygon | 69.9778783 | -151.2505431 |
| Arctic Peaty Lowland | Low-center polygon | 69.9731987 | -150.9298905 |
| Arctic Peaty Lowland | High-center polygon | 69.97252949 | -143.9911483 |
| Arctic Peaty Lowland | Low-center polygon | 69.96991956 | -152.1549267 |
| Arctic Peaty Lowland | Flat-center polygon | 69.96886667 | -147.4998772 |
| Arctic Peaty Lowland | Low-center polygon | 69.96768987 | -153.2857612 |
| Arctic Peaty Lowland | Low-center polygon | 69.96695899 | -151.3184863 |
| Arctic Peaty Lowland | Lake | 69.96656267 | -148.9642692 |
| Arctic Peaty Lowland | Low-center polygon | 69.96586765 | -148.9013185 |
| Arctic Peaty Lowland | Low-center polygon | 69.96289494 | -150.7278491 |
| Arctic Peaty Lowland | High-center polygon | 69.95771698 | -142.9332504 |
| Arctic Peaty Lowland | High-center polygon | 69.95702979 | -144.023177 |
| Arctic Peaty Lowland | River | 69.95525452 | -152.6473374 |
| Arctic Peaty Lowland | Low-center polygon | 69.95196917 | -148.9590192 |
| Arctic Peaty Lowland | High-center polygon | 69.95194883 | -151.5285351 |
| Arctic Peaty Lowland | non-patterned Drained Thaw Lake Basin | 69.94865459 | -151.146066 |
| Arctic Peaty Lowland | High-center polygon | 69.94638275 | -147.1084523 |
| Arctic Peaty Lowland | High-center polygon | 69.94434077 | -162.1822403 |
| Arctic Peaty Lowland | Low-center polygon | 69.94373038 | -146.5786528 |
| Arctic Peaty Lowland | High-center polygon | 69.93734574 | -146.7188935 |
| Arctic Peaty Lowland | Sandy barren | 69.92327072 | -144.055227 |
| Arctic Peaty Lowland | Drained slope | 69.92264234 | -147.2657149 |
| Arctic Peaty Lowland | High-center polygon | 69.92198651 | -147.2403995 |
| Arctic Peaty Lowland | Coastal saline water | 69.92167906 | -162.619836 |
| Arctic Peaty Lowland | Flat-center polygon | 69.92113483 | -161.841235 |
| Arctic Peaty Lowland | Drained slope | 69.92042287 | -150.9278259 |
| Arctic Peaty Lowland | Drained slope | 69.91834518 | -147.6378935 |
| Arctic Peaty Lowland | Drained slope | 69.91808622 | -142.6224465 |
| Arctic Peaty Lowland | River | 69.91767256 | -148.7023878 |
| Arctic Peaty Lowland | High-center polygon | 69.91589391 | -147.1844184 |
| Arctic Peaty Lowland | Drained slope | 69.91268354 | -143.4339201 |
| Arctic Peaty Lowland | River | 69.91108732 | -143.3781278 |
| Arctic Peaty Lowland | High-center polygon | 69.91050413 | -143.2878687 |
| Arctic Peaty Lowland | Flat-center polygon | 69.90860339 | -148.8817255 |
| Arctic Peaty Lowland | Sand dune | 69.90453006 | -149.5439561 |
| Arctic Peaty Lowland | Low-center polygon | 69.90397245 | -149.2292745 |
| Arctic Peaty Lowland | Drained slope | 69.90391229 | -150.9514025 |
| Arctic Peaty Lowland | High-center polygon | 69.90212503 | -147.5426711 |
| Arctic Peaty Lowland | High-center polygon | 69.89779713 | -151.4315359 |
| Arctic Peaty Lowland | High-center polygon | 69.89678649 | -162.2735406 |
| Arctic Peaty Lowland | Low-center polygon | 69.89306624 | -151.4679519 |
| Arctic Peaty Lowland | Flat-center polygon | 69.88652341 | -142.479606 |
| Arctic Peaty Lowland | High-center polygon | 69.88558837 | -147.4136194 |
| Arctic Peaty Lowland | Low-center polygon | 69.88449258 | -148.8607177 |
| Arctic Peaty Lowland | Low-center polygon | 69.87596818 | -147.4678887 |
| Arctic Peaty Lowland | High-center polygon | 69.86360973 | -147.5178127 |
| Arctic Peaty Lowland | Flat-center polygon | 69.86335799 | -142.3551036 |
| Arctic Peaty Lowland | High-center polygon | 69.85992471 | -154.6494156 |
| Arctic Peaty Lowland | High-center polygon | 69.85686164 | -162.2378945 |
| Arctic Peaty Lowland | non-patterned Drained Thaw Lake Basin | 69.85647126 | -154.5272207 |
| Arctic Peaty Lowland | Drained slope | 69.85594025 | -143.267407 |
| Arctic Peaty Lowland | High-center polygon | 69.85498966 | -147.9877972 |
| Arctic Peaty Lowland | Drained slope | 69.85456085 | -162.5223421 |
| Arctic Peaty Lowland | Low-center polygon | 69.84950904 | -143.5398509 |
| Arctic Peaty Lowland | High-center polygon | 69.84599955 | -150.8254797 |
| Arctic Peaty Lowland | Sandy barren | 69.84234766 | -152.0658642 |
| Arctic Peaty Lowland | Low-center polygon | 69.83791279 | -149.1262879 |
| Arctic Peaty Lowland | High-center polygon | 69.83593443 | -161.4918656 |
| Arctic Peaty Lowland | Low-center polygon | 69.83590834 | -142.9650343 |
| Arctic Peaty Lowland | Lake | 69.83156435 | -142.8805651 |
| Arctic Peaty Lowland | High-center polygon | 69.82653313 | -148.9383185 |
| Arctic Peaty Lowland | non-patterned Drained Thaw Lake Basin | 69.82532532 | -150.9021203 |
| Arctic Peaty Lowland | High-center polygon | 69.8240468 | -161.3155374 |
| Arctic Peaty Lowland | High-center polygon | 69.82383051 | -148.9568257 |
| Arctic Peaty Lowland | Low-center polygon | 69.8193974 | -148.0614951 |
| Arctic Peaty Lowland | High-center polygon | 69.81768683 | -148.0278702 |
| Arctic Peaty Lowland | Drained slope | 69.8175308 | -143.1050281 |
| Arctic Peaty Lowland | Riparian corridor | 69.81704761 | -148.7501113 |
| Arctic Peaty Lowland | High-center polygon | 69.81473929 | -161.6735509 |
| Arctic Peaty Lowland | Lake | 69.80835302 | -162.6923939 |
| Arctic Peaty Lowland | Drained slope | 69.80521034 | -142.8850841 |
| Arctic Peaty Lowland | Sandy barren | 69.80366986 | -141.898679 |
| Arctic Peaty Lowland | Low-center polygon | 69.79959212 | -148.7844455 |
| Arctic Peaty Lowland | Low-center polygon | 69.7995901 | -149.0413157 |
| Arctic Peaty Lowland | Flat-center polygon | 69.79906172 | -142.2861114 |
| Arctic Peaty Lowland | Sandy barren | 69.79901637 | -148.716765 |
| Arctic Peaty Lowland | Pond | 69.7957194 | -154.6411509 |
| Arctic Peaty Lowland | High-center polygon | 69.79473587 | -162.4293019 |
| Arctic Peaty Lowland | Coastal saline water | 69.79425912 | -162.948916 |
| Arctic Peaty Lowland | Drained slope | 69.79156 | -149.3837017 |
| Arctic Peaty Lowland | High-center polygon | 69.78976303 | -143.1523781 |
| Arctic Peaty Lowland | Low-center polygon | 69.7892051 | -162.563098 |
| Arctic Peaty Lowland | High-center polygon | 69.78630383 | -152.0087804 |
| Arctic Peaty Lowland | River | 69.78436149 | -149.6147959 |
| Arctic Peaty Lowland | Drained slope | 69.78268306 | -155.7648917 |
| Arctic Peaty Lowland | High-center polygon | 69.77910661 | -149.1117864 |
| Arctic Peaty Lowland | Drained slope | 69.77567493 | -150.9744488 |
| Arctic Peaty Lowland | High-center polygon | 69.77444569 | -150.7668476 |
| Arctic Peaty Lowland | non-patterned Drained Thaw Lake Basin | 69.77388775 | -149.3534449 |
| Arctic Peaty Lowland | Low-center polygon | 69.77245433 | -162.3470583 |
| Arctic Peaty Lowland | Sandy barren | 69.771133 | -142.3543467 |
| Arctic Peaty Lowland | Drained slope | 69.76790927 | -161.8635948 |
| Arctic Peaty Lowland | Low-center polygon | 69.76714971 | -142.8997372 |
| Arctic Peaty Lowland | High-center polygon | 69.76227518 | -152.1447148 |
| Arctic Peaty Lowland | High-center polygon | 69.76076231 | -148.8645564 |
| Arctic Peaty Lowland | non-patterned Drained Thaw Lake Basin | 69.75893243 | -151.140087 |
| Arctic Peaty Lowland | Sandy barren | 69.75712878 | -142.571874 |
| Arctic Peaty Lowland | Flat-center polygon | 69.75143609 | -162.5491427 |
| Arctic Peaty Lowland | High-center polygon | 69.74851834 | -149.4116787 |
| Arctic Peaty Lowland | High-center polygon | 69.74810287 | -161.961739 |
| Arctic Peaty Lowland | Riparian corridor | 69.74169533 | -149.0416871 |
| Arctic Peaty Lowland | Low-center polygon | 69.7389452 | -162.1920598 |
| Arctic Peaty Lowland | High-center polygon | 69.7372593 | -149.1480141 |
| Arctic Peaty Lowland | Low-center polygon | 69.73723101 | -161.9990934 |
| Arctic Peaty Lowland | High-center polygon | 69.73492562 | -152.1833446 |
| Arctic Peaty Lowland | Low-center polygon | 69.73111651 | -153.199321 |
| Arctic Peaty Lowland | Flat-center polygon | 69.72639788 | -141.9334881 |
| Arctic Peaty Lowland | High-center polygon | 69.72311494 | -162.7105922 |
| Arctic Peaty Lowland | High-center polygon | 69.72266983 | -149.62233 |
| Arctic Peaty Lowland | High-center polygon | 69.72085745 | -162.6515969 |
| Arctic Peaty Lowland | non-patterned Drained Thaw Lake Basin | 69.71781261 | -141.8290761 |
| Arctic Peaty Lowland | Low-center polygon | 69.71767378 | -142.0878945 |
| Arctic Peaty Lowland | Low-center polygon | 69.71663561 | -142.5600839 |
| Arctic Peaty Lowland | Low-center polygon | 69.71405814 | -154.5403982 |
| Arctic Peaty Lowland | High-center polygon | 69.70441333 | -162.289619 |
| Arctic Peaty Lowland | Low-center polygon | 69.70425308 | -162.6716817 |
| Arctic Peaty Lowland | Low-center polygon | 69.70019206 | -149.0739536 |
| Arctic Peaty Lowland | High-center polygon | 69.69925177 | -153.8652731 |
| Arctic Peaty Lowland | Low-center polygon | 69.69751779 | -162.3932144 |
| Arctic Peaty Lowland | non-patterned Drained Thaw Lake Basin | 69.68558266 | -162.716989 |
| Arctic Peaty Lowland | River | 69.68113393 | -151.4667765 |
| Arctic Peaty Lowland | High-center polygon | 69.67784461 | -149.6531341 |
| Arctic Peaty Lowland | non-patterned Drained Thaw Lake Basin | 69.67672028 | -162.5453526 |
| Arctic Peaty Lowland | Low-center polygon | 69.6765466 | -142.4257265 |
| Arctic Peaty Lowland | Low-center polygon | 69.67544441 | -142.8153736 |
| Arctic Peaty Lowland | High-center polygon | 69.67470882 | -148.7798461 |
| Arctic Peaty Lowland | High-center polygon | 69.67132573 | -141.6107393 |
| Arctic Peaty Lowland | Flat-center polygon | 69.67099925 | -141.7533687 |
| Arctic Peaty Lowland | non-patterned Drained Thaw Lake Basin | 69.66048723 | -162.0522267 |
| Arctic Peaty Lowland | Low-center polygon | 69.6602366 | -162.8717481 |
| Arctic Peaty Lowland | High-center polygon | 69.6561703 | -161.8758629 |
| Arctic Peaty Lowland | High-center polygon | 69.65324278 | -141.0891665 |
| Arctic Peaty Lowland | non-patterned Drained Thaw Lake Basin | 69.6532268 | -142.1615385 |
| Arctic Peaty Lowland | Low-center polygon | 69.64743296 | -148.7998935 |
| Arctic Peaty Lowland | Drained slope | 69.6452779 | -141.1748849 |
| Arctic Peaty Lowland | non-patterned Drained Thaw Lake Basin | 69.64274398 | -142.2652916 |
| Arctic Peaty Lowland | Low-center polygon | 69.6415219 | -141.8280441 |
| Arctic Peaty Lowland | Low-center polygon | 69.6410096 | -142.6527441 |
| Arctic Peaty Lowland | Drained slope | 69.64091987 | -151.2399589 |
| Arctic Peaty Lowland | non-patterned Drained Thaw Lake Basin | 69.63859149 | -148.8529098 |
| Arctic Peaty Lowland | Low-center polygon | 69.6250492 | -142.588875 |
| Arctic Peaty Lowland | High-center polygon | 69.6229068 | -141.0478011 |
| Arctic Peaty Lowland | High-center polygon | 69.6221047 | -141.5187006 |
| Arctic Peaty Lowland | Lake | 69.61507528 | -148.8241823 |
| Arctic Peaty Lowland | Sandy barren | 69.6124671 | -163.0720992 |
| Arctic Peaty Lowland | Low-center polygon | 69.60163759 | -148.8039905 |
| Arctic Peaty Lowland | High-center polygon | 69.59901001 | -141.9502703 |
| Arctic Peaty Lowland | High-center polygon | 69.59813182 | -151.4394728 |
| Arctic Peaty Lowland | High-center polygon | 69.5902851 | -154.3647156 |
| Arctic Peaty Lowland | High-center polygon | 69.5705555 | -162.9272247 |
| Arctic Peaty Lowland | Sandy barren | 69.56608018 | -151.4584285 |
| Arctic Peaty Lowland | High-center polygon | 69.56388068 | -162.979885 |
| Arctic Peaty Lowland | Sand dune | 69.53375961 | -148.5169008 |
| Arctic Peaty Lowland | High-center polygon | 69.53201653 | -162.4545666 |
| Arctic Peaty Lowland | High-center polygon | 69.52521864 | -162.9250437 |
| Arctic Peaty Lowland | Coastal saline water | 69.50114043 | -163.0668197 |
| Arctic Peaty Lowland | River | 69.48796992 | -162.4741614 |
| Arctic Peaty Lowland | Drained slope | 69.4478925 | -162.9714906 |
| Arctic Peaty Lowland | High-center polygon | 69.44723842 | -162.5483843 |
| Arctic Peaty Lowland | Drained slope | 69.40744651 | -162.6759297 |
| Arctic Peaty Lowland | Low-center polygon | 69.38862236 | -162.8341842 |
| Arctic Peaty Lowland | Lake | 69.38844234 | -162.9385939 |
| Arctic Peaty Lowland | Low-center polygon | 69.38530799 | -162.8199567 |
| Arctic Peaty Lowland | Drained slope | 69.37376615 | -163.0198029 |
